# Anti-Neuraminidase Bioactives from Manggis Hutan (*Garcinia celebica* L.) Leaves: Partial Purification and Molecular Characterization

**DOI:** 10.3390/molecules25040821

**Published:** 2020-02-13

**Authors:** Muchtaridi Muchtaridi, Milyadi Sugijanto, Amirah Mohd Gazzali, Habibah A. Wahab

**Affiliations:** 1 Department of Pharmaceutical Analysis and Medicinal Chemistry, Faculty of Pharmacy, Universitas Padjadjaran, Jl Raya 21.5 Bandung-Sumedang 45363, Indonesia; milyadi.sugijanto@yahoo.com; 2 Department of Pharmaceutical Technology, School of Pharmaceutical Sciences, Universiti Sains Malaysia, P Pinang 11800, Malaysia; amirahmg@usm.my; 3 Pharmaceutical Design and Simulation Laboratory, School of Pharmaceutical Sciences, Universiti Sains Malaysia, P Pinang 11800, Malaysia

**Keywords:** *Garcinia celebica*, catechin, friedeline, lanastone, molecular docking

## Abstract

The neuraminidase enzyme (NA) from the influenza virus is responsible for the proliferation and infections of the virus progeny, prompting several efforts to discover and optimize effective neuraminidase inhibitors. The main aim of this study is to discover a new potential neuraminidase inhibitor that comes from *Garcinia celebica* leaves (GCL). The bioassay-guided isolation method was performed to obtain lead compounds. The binding interaction of the isolated compounds was predicted by using molecular docking studies. Friedeline (GC1, log*P* > 5.0), two lanastone derivatives (methyl-3α,23-dihydroxy-17,14-friedolanstan-8,14,24-trien-26-oat (GC2) and 24E-3a,9,23-trihydroxy-17,14-friedolanostan-14,24-dien-26-oate (GC3) with Log*P* > 5.0) and catechin (GC4, Log*P* = 1.4) were identified. The inhibitory potency of these four compounds on NA from *C. perfringens* and H_1_N_1_ was found to be as follows: GC4 > GC2 > GC3 > GC1. All compounds exhibited higher inhibitory activity towards *C. perfringens* NA compared to H_1_N_1_ NA. From the molecular docking results, GC4 favorably docked and interacted with Arg118, Arg371, Arg292, Glu276 and Trp178 residues, whilst GC2 interacted with Arg118, Arg371, Arg292, Ile222, Arg224 and Ser246. GC3 interacted with Tyr406 only. GC4 had potent NA inhibition with free energy of binding of −12 kcal/mol. In the enzyme inhibition study, GC4 showed the highest activity with an IC_50_ of 60.3 µM and 91.0 µM for *C. perfringens* NA and H_1_N_1_ NA—respectively.

## 1. Introduction

Neuraminidase (NA) is an enzyme that plays an essential role in the cleavage of sialic acid from the terminal receptors of cells, which will subsequently release new viruses from infected cells. NA can be found in many families of viruses, bacteria, protozoa, some invertebrates and some mammalian cells [1,2]. NA from different organisms has a different binding affinity or substrate preference, but they have some structural similarities, with several conserved domains and amino acid residues at the binding site [3]. Generally, the NA of all organisms will cleave the ketosidic bonds between the oligosaccharides of glycoproteins or glycolipids and the non-reducing end of sialic acid. [4]. The NA of the influenza virus specifically hydrolyses α-2,3-sialic acid from a galactose moiety at the site active and less efficiently the α-2,6-sialic acid-galactocyl moiety [5].

NA from the influenza A virus can be classified into two genetically distinct groups [6]. Group 1 consists of N1, N4, N5 and N8 subtypes while Group 2 consists of N2, N3, N6, N7 and N9 subtypes. Group 1 has a 150-loop cavity adjacent to the active site that serves as a gateway for ligands to interact with NA [7]. This cavity is suitable as a potential binding site in the development of new anti-influenza drugs [8].

To date, new NA inhibitors have been developed through synthetic chemistry [9]. The utilization of bioactive compounds from natural products as starting materials is relatively unpopular, although this technique has a good potential and is relevant. Science has also shown that the combination of both methods is undoubtedly the most efficient way to accelerate the discovery of new and effective NA inhibitors. Oseltamivir for example, is synthesized from shikimic acid which cannot be obtained economically by synthesis, but can be efficiently isolated from Chinese star anise [10].

In our previously reported study, *Garcinia mangostana* (GM) showed potent NA inhibition on the H_5_N_1_ virus [11]. *Garcinia celebica* (GCL) is also from the same genus as *Garcinia mangostana* (family *Clusiaceae*) and is locally known as *manggis hutan* in the Island of Java. This plant, which is usually found in the forested area of the island [12,13], has a white sap and is poisonous whilst GM, widely cultivated, has a yellow sap and is non-toxic [14]. The flowers of GCL are aromatic as compared to other species [15]. Information about the biological activities of the GCL plant is not widely published. Among those reported are the antiplasmodial activity of triterpenoids from GCL leaves, which was published by Elfita and co-workers from Indonesia in 2009 [16]. In a relatively recent publication, another group of researchers, Subarnas et al. (2012), found that GCL is a good source of potential antiproliferative agents, that may be further developed into useful drug candidates. These limited publications indicate that GCL is potentially useful and should be explored.

Due to the fact that GCL also belongs to the same *Clusiaceae* family as GM, the present study aimed to isolate and characterize compounds from GCL that may have potential as a NA inhibitor by the bioassay-guided isolation method [17]. For the purpose of this study, leaves of GCL were selected instead of the fruits due to the fact that its fruits are not easily available, because of consumption by wild animals [18]. The extracts of the leaves were tested against a NA enzyme using 2’-(4-methylumbelliferyl)-α-D-*N*-acetylneuraminic acid (MUNANA) assay, and the active extracts were further fractionated to isolate pure compounds. Four compounds, friedeline, two lanastone derivatives (methyl-3α, 23-dihydroxy-17,14-friedolanstan-8,14,24-trien-26-oat and 24E-3a,9,23-trihydroxy-17,14-friedolanostan-14,24-dien-26-oate) and cathechin were isolated and their NA inhibition activity was evaluated through a MUNANA assay. Their binding interaction was then predicted through molecular docking simulation.

## 2. Results

### 2.1. Extraction, Isolation and Bioassay

**Figure 1:**
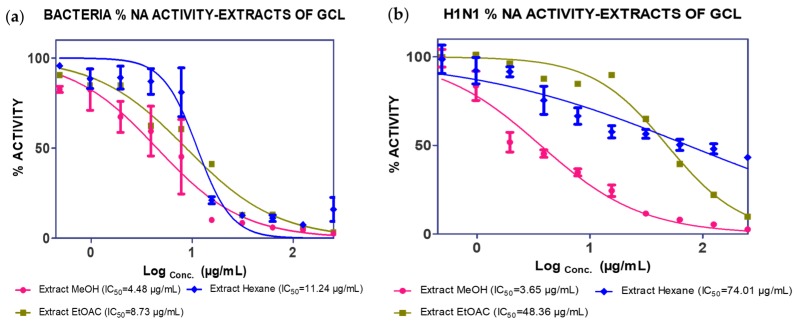
Neuraminidase enzyme (NA) inhibition activity of *Garcinia celebica* leaves (GCL) extracts against (**a**) *C. perfringens*-NA and (**b**) H_1_N_1_-NA.

The MeOH extract was tested against *C. perfringens* NA and an IC_50_ value of 4.84 µg/mL was recorded. The EtOAc extract showed activity against *C. perfringens* NA (8.73 µg/mL) and H_1_N_1_ NA (48.36 µg/mL). The *n*-hexane extract was also found to inhibit both *C. perfringens* and H_1_N_1_ NA (Figure 1).

**Figure 2:**
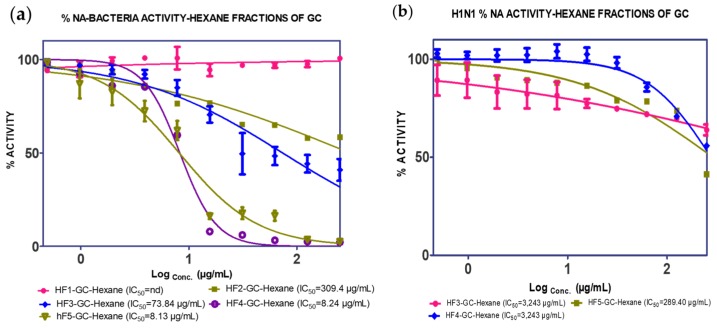
NA inhibition activity of *n*-hexane fractions of GCL against (**a**) *C. perfringens*–NA and (**b**) H_1_N_1_-NA.

The extracts of *n*-hexane and EtOAc were further fractionated to enable the identification of specific active compounds. Fractionation of the *n*-hexane extract gave five fractions (F1–F5). F3, F4 and F5 showed the ability to inhibit both *C. perfringens* NA and H_1_N_1_-NA, as shown in Figure 2.

F3, F4 and F5 were further fractionated to obtain pure compounds. Friedeline (labelled as GC1) was obtained from F3 (101.5 mg) and the identification of this compound was in concordance with several other previously reported studies (see Supplementary Materials) [19,20,21]. Then, (24E)-3a, 9, 23-trihydroxy-17,14-friedolanostan-14,24-dien-26-oate labelled as GC2 (25.4 mg) were isolated from F4 and the structural profile is similar, as reported by Rukachaisirikul et al. (2000). Methyl-3α,23-dihydroxy-17,14-friedolanstan-8,14,24-trien-26-oat (labelled as GC3) was isolated from F4 (11.1 mg) and F5 (32.1 mg). The spectroscopy analysis of the third compound (methyl-3α,23-dihydroxy-17,14-friedolanstan-8,14,24-trien-26-oat) confirmed the identity of the compound, based on data from the previous study (see Supplementary Materials) [22].

**Figure 3:**
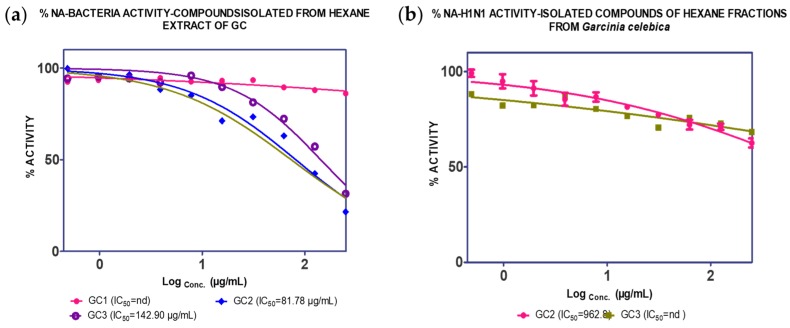
Inhibition activity of isolated compounds from *n*-hexane fraction of GCL against (**a**) *C. perfringens*–NA and (**b**) H_1_N_1_-NA.

As illustrated in Figure 3, GC1 was not active against *C. perfringens*-NA, whilst GC2 and GC3 [23] showed inhibition against *C. perfringens*-NA with a maximum inhibition of 79% (IC_50_ = 81.78 µg/mL) and 62% (IC_50_ = 142.90 µg/mL), respectively (Figure 3). With regards to the activity on H_1_N_1_-NA, both GC2 and GC3 did not show any significant activity.

**Figure 4:**
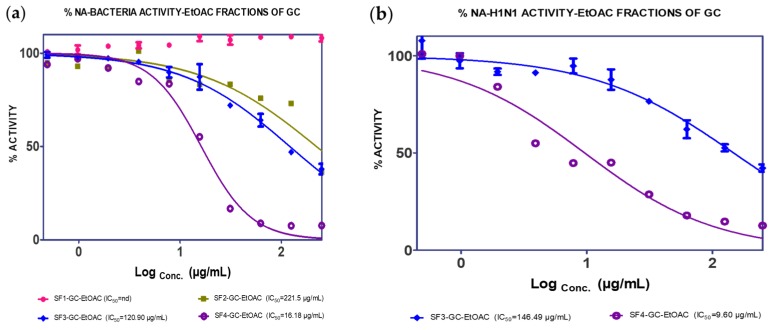
Neuraminidase inhibition activity of EtOAc fractions of GCL against (**a**) *C. perfringens*–NA and (**b**) H_1_N_1_-NA.

**Figure 5:**
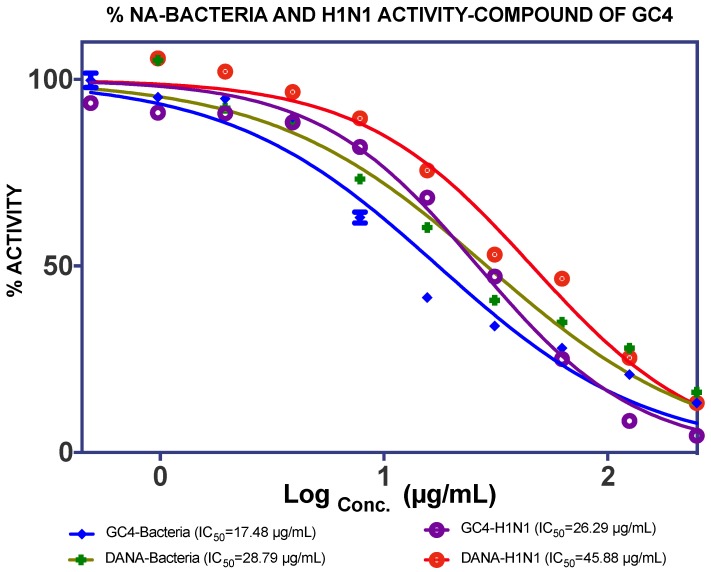
NA inhibition of isolated compound catechin as compared to DANA, as a gold standard against *C. Perfringens*-NA (blue) and H_1_N_1_-NA (purple).

The EtOAc extract was found to be more active against NA than the *n*-hexane extract. Fractionation of this extract (19.69 g) gave four fractions (SF1: 0.9 g, SF2: 5.2 g, SF3: 2.8 g, SF4: 6.1 g), and it was found that SF4 showed good IC_50_ values against *C. perfringens*–NA and H_1_N_1_-NA, as shown in Figure 4. Subsequently, GC4 was isolated from this fraction (12.8 mg). A spectroscopy analysis of GC4 confirmed that GC4 is a compound called catechin. This compound showed good NA inhibition ability with the IC_50_ of 60.29 µM for *C. perfringens*–NA and 90.59 µM for H_1_N_1_-NA respectively, as shown in Figure 5.

### 2.2. Binding Interaction of the Isolated Compound from Garcinia celebica

As shown in Table 1, GC1 was found to be inactive against NA and this may be attributed to the absence of a hydrogen bond donor, such as hydroxyl moieties, in the molecular structure. This finding was parallel to the result of the molecular docking study, in which GC1 was found to have low docking favorability in NA. This will be further explained in the following subsection.

The compounds GC2 and GC3 are friedolanostane derivatives. The presence of these compounds in *Garcinia* sp. has been reported previously in the literature [24,25]. Viera et al. (2004) reported on the isolation of 11 friedolanostane-related compounds from *Garcinia speciosa* leaves [26]. Five other friedolanostanes were isolated by Rukachaisirikul et al. (2005) from *Garcinia hombroniana* leaves [22], whilst two friedolanostane compounds were reported by Klaiklay et al. (2013) from the twigs of *Garcinia hombroniana* [24]. Nguyen et al. (2011), on the other hand, reported on the isolation of eight friedolanostane compounds from *Garcinia benthami* bark and leaves [27].

**Figure 6:**
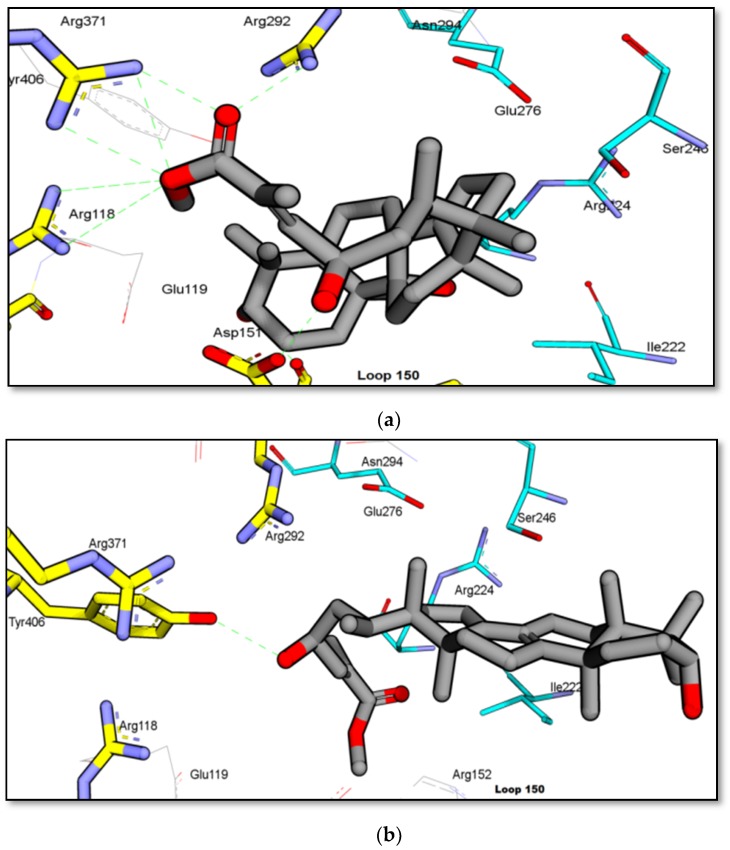
Binding interaction of isolated compounds (**a**) GC2 and (**b**) GC3 from GCL against H_1_N_1_–NA (PDB code: 3B7E). (blue carbon: hydrophobic residues).

The ester functional group present in GC2 and GC3 might play an important role in increasing the activity of the molecules on NA. Experimentally, GC3 showed an IC_50_ of more than 100 µg/mL. In the molecular docking study, the skeleton of GC3 (ring A, B, and C) was found to be positioned close to the hydrophobic pocket, as shown in Figure 6b. GC3 formed a hydrogen bond between 23-OH (from GC3) and Tyr406 from the enzyme, and did not form any interaction with the arginine triad. Thus, this compound is expected to be less active than the isolated flavonoid.

GC2 showed a better interaction with NA as compared to GC3. It docked well with a free energy of binding, FEB of −10 kcal/mol. The presence of hydroxyl group at C-9 made the skeleton of triterpene more flexible. The ester group of GC2 interacted well with the arginine triad, as shown in Figure 6a. Two oxygens from the ester group accepted protons from Arg118, Arg371, and Arg292, while the 23-OH moiety donated a proton to Asp151 from Loop150. In addition, ring B and C of GC2 were positioned close to the hydrophobic pocket (Ile222, Arg224, and Ser246) and this is the reason why GC2 has the lowest FEB and high fit value to map with T2S202 model. Unfortunately, the activity of GC2 on *C. perfringens*-NA was classified as less active (IC_50_ 81.72 µg/mL or 123.26 µM). Similarly, as implied from the experimental IC_50_, the GC2 activity against H_1_N_1_-NA was not as good as the predicted activity based on the free energy of binding. One possible reason for this is the low solubility of this compound, which might have inversely affected the bioassay result. In drug discovery and development, the solubility of active compounds has a big influence on the administration, distribution, metabolism and excretion (ADME) characteristics of a particular compound [28,29]. Based on Table 2, LogP of GC2 and GC3 were 5.18 and 6.14 respectively, as predicted by the software (DS 2.5), and thus, they were categorized as having “poor” drug-like properties. Lipiski et al. (2012) [30] predicted that poor absorption or permeation is more likely once logP is greater than five [31,32]. In this study, GC2 and GC3 were dissolved in a slightly higher concentration of DMSO (2.5%), due to low solubility and precipitation that might have occurred when the MES buffer was added. Poor solubility may also cause other problems, including poor bioavailability in oral administration, difficulty in formulation, lack of efficacy, high toxicity, expensive and prolonged development, and the need for multiple daily doses [28,29].

Catechin or GC4 was found to be the most active as a NA inhibitor, compared to the other three isolated compounds and DANA (2,3-didehydro-2-deoxy-*N*-acetylneuraminic acid), as a commercial inhibitor. The IC_50_ of GC4 against *C. perfringens*-NA and H_1_N_1_-NA were 17.48 µg/mL (60.27 µM) and 26.29 µg/mL (90.95 µM) respectively and, thus, this compound can be classified as moderately active.

**Figure 7:**
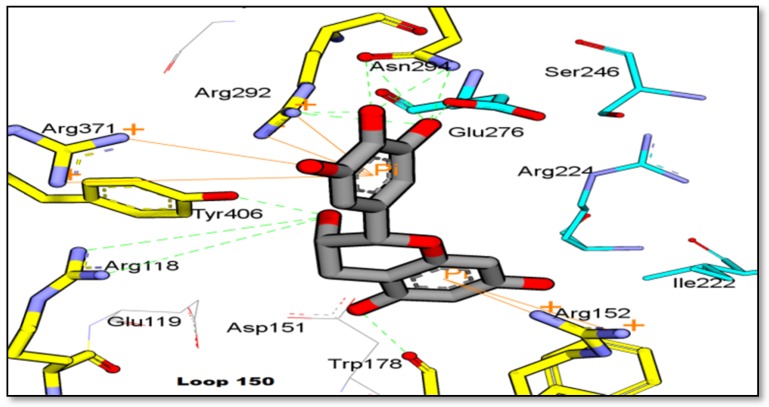
Binding interaction of GC4 from GCL against H_1_N_1_-NA (PDB code: 3B7E). (Blue carbon: hydrophobic residues).

In this study, the molecular interaction of catechin and H_1_N_1_-NA (PDB code: 3B7E [33]) was investigated. Catechin favorably docked onto NA at the 2-catechol moiety (ring C), and it interacted well with the arginine triad through hydrogen bond and *pi*-cation interactions with binding energy –12 kcal/mol. As shown in Figure 7, it appeared that the compound did not form any interaction with the hydrophobic pocket (Ileu222, Arg224, and Ser246), but interacted through hydrogen bonding with Glu276. The 3-OH moiety of catechin formed a hydrogen bond with Trp178 (2.3 Å) and it is linked to 3-gallocyl to form epicatechin gallate (ECG).

## 3. Discussion

Four compounds were isolated from GCL as listed in Table 1. They were obtained based on the results of NA inhibition by fractions recuperated from GCL extracts. Two of the three triterpenoids showed satisfactory inhibition against *C. perfringens*-NA, but they were less active against H_1_N_1_-NA (GC2 and GC3). This may be attributed to the nature of *n*-hexane extracts (hydrophobic molecules), which usually showed low activity against NA because of their low water solubility characteristics. Solubility is a very important factor that influences the inhibitory activity of compounds against NA [34]. Although this factor was recognized as a limitation in this current study, the discovery of active triterpenoids from GCL has never been reported before and this is an important finding that needs to be recorded and reported. The activity of these triterpenoids against NA is an important finding that could lead to the development of new actives through further simulations and synthetic chemistry.

GC4 showed moderate activity against both *C. perfringens*-NA and H_1_N_1_-NA. This flavonoid was confirmed as catechin and was obtained from EtOAc extracts. This compound was obtained from the fractionation results, and found that F4, which has inhibitory activity against NA, was best compared to other fractions. Catechin was already being reported as present in some *Garcinia* sp. such as *G. kola* [35] and *G. penangiana* [36]. However, to the best of the authors’ knowledge, there are no reports precising the presence of this compound in *G. celebica*. Catechin was previously evaluated in vitro for its anti-influenza properties, and it showed good inhibition of influenza virus replication [37,38]. Another group of researchers investigated the ability of catechin-containing herbal tea to halt influenza virus infection in residents of a nursing home for the elderly, and they reported positive results [39].

Kuzuhara et al. (2009) in their publication explained that catechin inhibited the endonuclease activity of RNA polymerase in influenza A virus, thus this compound has a big potential to be further developed as an anti-influenza A drug [40]. Its action against the influenza virus could also be attributed to its antioxidant property. Liu et al. (2008) have discussed the anti-influenza activity of catechin, but the mechanism of action of this molecule at molecular level was not investigated [41]. Shan et al. (2012) proposed that the 4-chromanone moiety in catechin is responsible for its NA inhibition activity [42]. Uchide and Toyoda (2011) noted that the activity of ECG as an influenza virus inhibitor is contributed to mainly by the 3-gallocyl moiety of this compound, whereas the 5’-OH at the trihydroxybenzyl moiety at the 2-position plays a minor role. The presence of the hydroxyl group on C-5′ played a critical role in the inhibition of NA [43].

The antioxidant property of catechin means that this molecule could scavenge the superoxide anion and hydroxyl radicals [44]. The orientation of the 4-chrommanone ring allowed catechol moiety to rotate, thus it could interact with the triad arginine residues (Asp151, Arg 292 and Arg 371). Three arginine residues (Arg 118, Arg 292, and Arg 371) and a glutamate residue (Glu 276) have an important role in the binding of sialic acid in the active site of NA [45]. These results are in line with a study conducted by Muller et al. [46], in which the phenyl ring of 4-chromanone moiety was favored by the Ile427 and Lys432 residues that formed the hydrophobic pocket of NA. However, this was not seen in the molecular docking conducted in this study, and instead it appeared to interact with Ileu222, Arg224, and Ser246 as the hydrophobic pocket.

## 4. Materials and Methods

### 4.1. Plant Materials

The leaves of *Garcinia celebica* (*G. celebica*) were collected from Pangandaran, West Java, Indonesia in July 2011 (voucher specimen no. 112/HB/7/2011). Dried leaves powder of *G. celebica* or *manggis hutan* (1.0 kg) were macerated with methanol (1:3 *w*/*v*, three times, 5 L, for 24 h each time).

The solvent was evaporated under reduced pressure to yield a concentrated methanol extract (179.8 g). The mixture of MeOH-water was filtered and further partitioned with *n*-hexane and EtOAc successively to give *n*-hexane and EtOAc fractions (26.9 g and 46.5 g, respectively).

### 4.2. Isolation of Compounds from n-Hexane Extract of GCL

The *n*-hexane extract (4.6 g) was fractionated through gravity column chromatography (2 × 30 cm), by using *n*-hexane/EtOAc as solvents, to afford five fractions. From these five fractions, F3, F4 and F5 gave good NA inhibition against *C. perfringens* NA (more than 50% inhibition), and thus, these fractions were further purified to isolate the active compounds. The F3 fraction (987.8 mg) was dissolved in hexane and white crystalline needles precipitated. These crystals were re-crystallized in CHCl_3_/MeOH (1:3) to obtain pure crystals, GC1 (101.5 mg). The F4 fraction (896.1 mg) was purified through column chromatography with the mixture of CHCl_2_/*n*-hexane/MeOH (6.5:3:0.5 and 7:2.5:0.5) as a solvent system to afford GC2 (white powder, 25.4 mg), and GC3 (yellow powder, 11.1 mg). The F5 fraction was subjected to Preparative Layer Plates Chromatograpy (PLC) with *n*-hexane/MeOH solvent system (6.5:3:0.5) to afford GC3 (32.1 mg).

### 4.3. Isolation of Compounds from EtOAc Extract of GCL

The crude extract from EtOAc (19.7 g) was subjected to gravity column chromatography (5 × 30 cm) with CHCl_3_/MeOH in a stepwise manner at 10%, producing four fractions (SF1, SF2, SF3, and SF4). The fractions were assayed for their NA inhibition, and F3 and F4 were selected because of their good activity (more than 50% inhibition) against *C. perfringens* NA. Further assay works with H_1_N_1_ NA were conducted, in which SF4 showed up to 90% inhibition. SF4 (2.6 g) was subsequently subjected to small column chromatography (1 × 20 cm) with a CHCl_3_/MeOH (88:12) solvent system, to produce 76 fractions. Fraction 39–42 (831.1 mg) was further purified through semi-preparative liquid chromatography (three times) to obtain GC4 (12.8 mg).

### 4.4. General Experiments and Spectroscopy Methods

^1^H NMR and ^13^C NMR spectra were both recorded with a BRUKER AVANCE III 500 MHz spectrometer. Mass spectra were measured on an Agilent 1100 Series LC-MSD-Trap-VL spectrometer by using electrospray ionisation as the type of ion source. FTIR spectra were recorded using an IR-Prestige-21 (Shimadzu) spectrometer. Melting points were obtained by using an electrothermal melting point apparatus (STUART-SMP10). UV spectra were determined on an UV-Vis spectrophotometer (Analytical Jena, specord-200). Rotation index was determined using ADP 120 Bellingham (Stanley Ltd., Tokyo, Japan) The complete spectral data is provided in Supplementary Materials.

### 4.5. Neuraminidase (NA) Activity

NA was prepared in 2-(*N*-morpholino) ethanesulfonic acid (MES) buffer (Sigma^®^) to get a concentration of 0.3 µ/mL. The substrate MUNANA was prepared in the same buffer to get a concentration of 100 μM. The *G. celebica* leaves’ extracts, fractions, and isolated compounds were prepared in 2.5% DMSO (Merck^®^), due to the solubility problem in concentrations between 7.8125 to 125 µg/mL. The times of incubation (agitated at 200 rpm, 37 °C) for the mixture of NA-coffee and NA-coffee-MUNANA were 30 min and 60 min, respectively, and the reaction was stopped by using glycine before reading. NA activity towards inhibitors was measured via a fluorogenic substrate, MUNANA, excited at 365 nm, with fluorescence emission at 450 nm, by using an ELISA microplate reader (Tecan-i-control infinite 200Pro) [47]. The data results were analyzed by GraphPad Prism 5.0.

### 4.6. Molecular Docking Simulation

Molecular docking methods were adopted from the previous study [48]. The NA protein of subtype N1 in complex with zanamivir (PDB code: 3B7E [33]) was used as the target. Molecular docking simulations were performed with AutoDock 4.2 [49].

## 5. Conclusions

Friedeline, catechin and two lanastone derivatives (methyl-3α, 23-dihydroxy-17,14-friedolanstan-8,14,24-trien-26-oat and 24E-3a,9,23-trihydroxy-17,14-friedolanostan-14,24-dien-26-oate) were obtained from *G. celebica* leaves by using bioassay-guided isolation. Based on the enzyme inhibition study, the two lanastone derivatives showed low activity on NA while friedeline was inactive. Catechin, on the other hand, showed the highest activity as a NA inhibitor compared to the other three compounds. On the contrary, a molecular docking study showed that the two lanastone derivatives have a good docking profile on the binding site of NA. This may be due to the solubility problem as discussed earlier, which may have inversely affected the assay performance of the compounds. Another possible reason may be the fact that although the compounds docked well, they may not have had sufficient time to exert their inhibitory effect on the enzyme, hence the IC_50_ values of these compounds were high. From this study, it is suggested that the development of catechin as an anti-influenza agent would be valuable, but further structure modification may be needed to improve its inhibition activity.

## Figures and Tables

**Table 1:** Highlight of bioassay-guided isolation for NA inhibitors from GCL.

Extract	Partitions	Fractions	Compounds	IC_50_ *	
				**NA-*C. perfringens*^a^**	**NA-H1N1 ^b^**
MeOH extract				4.48 µg/mL	3.65 µg/mL
(17.98% mass)	Hexane extract			11.04 µg/mL	74.01 µg/mL
	(14.96% *w*/*w*)	F1 (5.07%)		nd	nd
		F2 (4.5%)		39.42 µg/mL	nd
		F3 (21.40%)		73.84 µg/mL	3.243 µg/mL
			GC1 (2.20%)	*nd*	nd
		F4 (21.28%)		8.24 µg/mL	289.4 µg/mL
			GC2 (0.55%)	81.74 µg/mL	962.80 µg/mL
			GC3 (0.24%)	142.49 µg/mL	nd
		F5 (21.45%)		277.0 µg/mL	nd
	EtOAc extract			38.39 µg/mL	48.36 µg/mL
	(25.86% *w*/*w*)	F1 (4.80%)		nd	nd
		F2 (26.39%)		120.90 µg/mL	nd
		F3 (14.21%)		221.50 µg/mL	146.49 µg/m
		F4 (30.4%)		16.18 µg/mL	9.60 µg/mL
			GC4 (0.49%)	17.48 µg/mL	26.29 µg/mL

^a^ IC_50_ of samples against *C. perfringens*-NA; ^b^ IC_50_ of samples against H_1_N_1_-NA; * nd; no detection.

**Table 2:** Data on the structures, physicochemical properties, and Lipinski’s rule of five of the isolated compounds.

Compound Code	Molecular Structure	Molecular Formula	MW	HBD	HBA	Log *P*
GC1	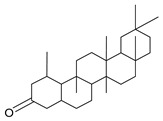	C_30_H_50_O	426	1	3	7.03
GC2	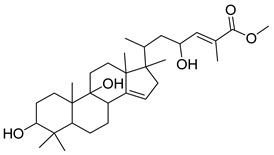	C_31_H_48_O_4_	502	3	5	5.18
GC3	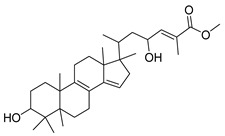	C_31_H_48_O_4_	485	2	4	6.13
GC4	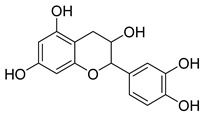	C_15_H_14_O_6_	290	5	6	1.5

MW: Molecular Weight; HBD: Hydrogen Bond Donor; HBA; Hydrogen Bond Receptor.

## Supplementary Materials

The following are available online, Figure S1: Spectral Data, Figure S2: Isolation Methods, Melting points and Spectral data of GC1-GC4.
