# Supplementary material for: Anti-Neuraminidase Bioactives from Manggis Hutan (Garcinia celebica L.) Leaves: Partial Purification and Molecular Characterization
Source: Molecules. 2020 Feb 13;25(4):821. doi: 10.3390/molecules25040821 (PMC7070733; doi:10.3390/molecules25040821)
Supplement: Supplementary file 1 [file molecules-25-00821-s001.zip › SUPPLEMENTARY A.docx]

**SUPPLEMENTARY A: METHOD OF ISOLATION AND SPECTRAL DATA**

**Isolation of Compounds from Hexane Extract of *G. celebica***

Hexane extract (4.6 g) was subjected to gravitation column chromatography (2 x 30 cm) using hexane-EtOAc as solvents (85:15 for 600 mL, 80:20 for 200 mL, and 70:30 for 200 mL) to afford five fractions [HF1 (233.3 mg), HF2 (208.1 mg), HF3 (987.8 mg), HF4 (979.0 mg), and HF5 (987.1)]. HF3, HF4 and HF5 gave good neuraminidase inhibition against bacteria-NA, thus it was subsequently fractioned to find the active compounds.

HF3 were washed with hexane and ~~to obtain~~ white crystalline needle was obtained which was GC1 (101.5 mg). GC1 was re-crystallized by CHCl_3_-MeOH (1:3). HF4 (896.1 mg) was subjected to column chromatography with DCM-Hexane-MeOH solvent system (6.5:3:0.5 (200 ml) and 7:2.5:0.5 (200 ml)) to afford GC2 (25.4 mg ). From HF4, yellow compound GC3 (11.1 mg) was obtained using Preparative Liquid Chromatography (PLC) with DCM-Hexane-MeOH solvent system (6.5:3:0.5). Fraction HF5 was subjected to PLC with Hexane-MeOH solvent system [6.5:3:0.5 (200 ml)] to afford GC4 (32.1 mg).

**Spectral Data of Friedeline (GC1)**

GC1 appeared as white crystalline needles with melting point 255-260^o^C (reference 360-263^o^C, ([Klass et al. 1992](#_ENREF_14))). It was characterised as follows: UV[(*n*-hexane), λ_max_] at 223 nm and 260 nm. IR-max cm^-1^: 2929, 2869 (C=C streching), 1715 (C=O), 1388, 1203. GC-MS (ESI) calculated for C_30_H_50_O [M+H^+^]: *m/z* 427.7242, found 426. ^1^H NMR (500 MHz, CDCl_3_) δ ppm 0.75 (s, 3H, H-24) 0.81 (s, 1 H, H-25) 0.88 (s, 3 H, d, J=6.5, H-23) 0.98 (s, 3 H-30) 1.02 (s, 3 H, H-26) 1.03 (3H, s, H-29) 1.07 (s,3H, H-27) 1.32 (m, 2H, H-12) 2.21-2.25 (m, 2H, H-7) 2.25 (q, *J*=6.1, 1 H, H-4) 2.43 ((dd, *J*=5.12, 1.97 Hz, 2 H, H-2) (. ^13^C NMR (500 MHz, CDCl_3_) δ ppm 6.82 (s, C-23) 14.66 (s, C-24) 17.95 (s, C-25) 18.25 (s, C-7) 18.66 (s, C-27) 20.26 (s,C-26) 28.18 (s, C-20) 30.00 (s, C-17) 30.51 (s, 2 C) *31.79 (s, C-28*) 32.10 (s, C-28) 32.43 (s, 15) 32.78 (s, 2 C) 35.02 (s, C-29) 35.36 (s, C-29) 35.64 (s, C-19) 36.02 (s, C-16) 37.46 (s, C-9) 38.31 (s, C-14) 39.26 (s, C-22) 39.71 (s, C-13) 41.31 (s, C-6) 41.53 (s, C-2) 42.15 (s, C-5) 42.81 (s, C-18) 53.12 (s, C-18) 58.24 (s, C-4) 59.49 (s, C-10) 213.23 (s, C-3).

**Spectral Data of Methyl (24E)-3a,9,23-trihydroxy-17,14-frie­dolanostan-14,24-dien-26-oate (GC2)**

GC2 appeared as white solid with melting point 130-135^o^C (reference 128-130^o^C,([Rukachaisirikul, et al. 2000](#_ENREF_26))); $[{\alpha]}_{D}^{25}$ =-100° (c=1, MeOH); UV[(MeOH), λ_max_] at 230 nm. IR-max cm^-1^: 3459 (br, OH), 2929 (C=C streching), 1705 (C=O). MS (ESI) calculated for C_31_H_48_O_4_ [M+H]^+^: *m/z* 485.71, found 502.3 (in the presence of one water molecule). ^1^H NMR (500 MHz, CDCl_3_) δ ppm 0.70 (6H s Me-18), 0.78 (3H, s, Me-21), 0.85 (3H, d, J=7.0 Hz, Me-21), 0.89 (3H, s, Me-28), 0.91 (6H s Me-19), 1.01-1.10 (2H, m, H-1, H-22), 1.19 (3H, s, Me-30), 1.45-1.50 (2H, m, H-6, H-7), 1.05-1.56 (1H, m, H-22), 1.54-1.47 (2H, m, H-6, H-12), 1.32-1.40 (2H, m, H-1, H­2), 1.53-1.67 (3H, m, H-2, H-11, H-12), 1.80 (3H, d, J=1.1 Hz, Me-27), 1.83-1.75 (2H, m, H­11, H-16), 1.90-2.20 (2H, m, H-5, H-7), 2.04-2.32 (1H, m, H-16), 2.15-2.25 (1H, m, H-20), 2.19-2.31 (1H, m, H-8), 3.32 (1H, brs, H-3), 3.75 (3H, s, OMe), 4.49 (1H, ddd, J=10.7, 7.2 and 2.5 Hz, H-23), 5.26 (1H, brm, H-15), 6.70 (1H, qd, J=8.2 and 1.4 Hz, H-24). ^13^C NMR (500 MHz, CDCl_3_) δ ppm 12.73 (C-27), 15.10 (C-21), 15.36 (C-18), 16.36 (C-19), 19.44 (C-30), 20.80 (C-6), 22.02 (C-29), 23.63 (C-1), 25.12 (C-2), 25.71 (C-7), 28.48 (C­28), 29.02 (C-12), 29.66 (C-11), 32.99 (C-20), 37.53 (C-4), 39.0 6(C-5), 39.14 (C-22), 39.17 (C-8), 42.19 (C-10), 44.74 (C-16), 49.09 (C-13), 51.95 (OMe), 54.02 (C-17), 66.81 (C-23), 75.43 (C-9), 76.12 (C-3), 120.45 (C-15), 127.14 (C-25), 144.39 (C­24), 153.35 (C-14), 168.5 (C-26).

**Spectral Data of Methyl-3α, 23-dihydroxy-17,14-friedolanstan-8,14,24-trien-26-oat** **(GC3)**

GC3 appeared as white powder with melting point 112-113^o^C (reference 112-113^o^C ([Rukachaisirikul, et al. 2000](#_ENREF_26))); $[{\alpha]}_{D}^{25}$ =-125° (c=1, MeOH); UV[(MeOH), λ_max_ ] at 275, 439 nm. IRmax cm^-1^: 3436 (br, OH), 2969 (C=C streching), 1705 (C=O). MS (ESI) calculated for C_31_H_48_O_4_ [M+H]^+^: *m/z* 485.71, found 485.254. ^1^H NMR (500 MHz, CDCl_3_) δ ppm 0.68 (6H s Me-18), 0.78 (3H, s, Me-21), 0.87 (3H, d, J=7.5 Hz, Me-21), 0.92 (3H, s, Me-28), 0.84 (6H s Me-19), 1.97-2.02 (2H, m, H-1, H-22), 0.94 (3H, s, Me-30), 2.14-2.17 (2H, m, H-7), 1.05-1.56 (1H, m, H-22), 1.19-1.23 (2H, m, H-6, H-12), 1.50-1.60 (3H, m, H-1, H-2, H-5, H-12), 1.63-1.73 (9H, m, 2H), 1.80 (3H, d, J=1.5 Hz, Me-27), 2.24-2.30; 1.99-1.94 (1H, m, H-16), 2.14-2.17 (2H, m, H-5, H-7), 2.24-2.30; 1.99-1.94 (1H, m, H-16), 2.12 (H-20 2H, m, H-20), 3.37 (1H, brs, H-3), 3.69 (3H, s, OMe), 4.48 (1H, ddd, J=10.7, 7.5, 2.5 Hz, H-23), 5.20 (1H, brs, H-15), 6.63 (1H, qd, J=10.7, 7.5 and 2.5 Hz, H-24). ^13^C NMR (500 MHz, CDCl_3_) δ ppm 12.74 (C-27), 15.27 (C-21), 15.65 (C-18), 17.09 (C-19), 18.15 (C-6), 18.95 (C-30), 22.19 (C-29), 22.73 (C-22), 25.60 (C-12), 26.70 (C-7), 27.99 (C­28), 29.23 (C-2), 30.01 (C-1), 33.41 (C-20), 37.60 (C-4), 37.82 (C-10), 39.47 (C-11), 44.46 (C-5), 45.55 (C-16), 48.02 (C-13), 50.04 (C-17), 51.95 (OMe), 66.90 (C-23), 75.86 (C-3), 115.81 (C-15), 122.86 (C-8), 127.13 (C-25), 142.37 (C­24), 144.41 (C-9),148.80 (C-14), 168.48 (C-26).

**Spectral Data of Catechin (GC4)**

GC4 appeared as brownish dark amorphous solid with melting point 178-180^o^C; $[{\alpha]}_{D}^{25}$=+15° (c=1, acetone); UV[(MeOH), λ_max_ ] at 277, 326 nm. IR-max cm^_1^: 3436 (br, OH), 2969 (C=C streching), 1705 (C=O). HRMS (QTOF) calculated for C_15_H_14_O_6_ [M+H] ^+^: *m/z* 291.27, found 291.0811. ^1^H NMR (500 MHz, MeOD) δ ppm 2.74 (d, *J*=2.84 Hz, 1 H), 2.74 (d, J=16.75, 3 Hz, 1 H, H-4a), 2.87 (d, *J*=4.57 Hz, 1H, H-4b), 4.12 (tt, J=7.10 Hz, 1H, H-4), 4.60 (d, J=7.45 Hz, 1 H, H-2), 5.96 (d, *J*=2.21 Hz, 5 H, H-8), 6.77 (d, J=8.15 Hz, 1 H, H-5’), 6.80 (d, J=1.91 Hz, 1 H, H-2’), 6.82 (dd, J=8.13, 1.98 Hz, 1 H, H-6’). ^13^C NMR (126 MHz, MeOD) δ ppm 29.28 (C-4), 67.51 (C-3), 79.91 (C-2), 96.42 (C-6), 100.09 (C-8, C-10), 115.35 (C-2’), 115.91 (C-5’) 119.41 (C-6’) 132.31 (C-1’) 145.81 (C-4’) 145.98 (C-3’) 157.40 (C-9) 157.71 (C-5) 158.03 (C-7).
