# Supplementary material for: Anti-Neuraminidase Bioactives from Manggis Hutan (Garcinia celebica L.) Leaves: Partial Purification and Molecular Characterization
Source: Molecules. 2020 Feb 13;25(4):821. doi: 10.3390/molecules25040821 (PMC7070733; doi:10.3390/molecules25040821)
Supplement: Supplementary file 1 [file molecules-25-00821-s001.zip › Supplementary file B-Sepctral data.docx]

**SUPPLEMENTARY B: SPECTRAL FIGURES DATA**

**Spectroscopy data of GC1**

IR spectrum of GC1 compound that isolated from *G. celebica* leaves


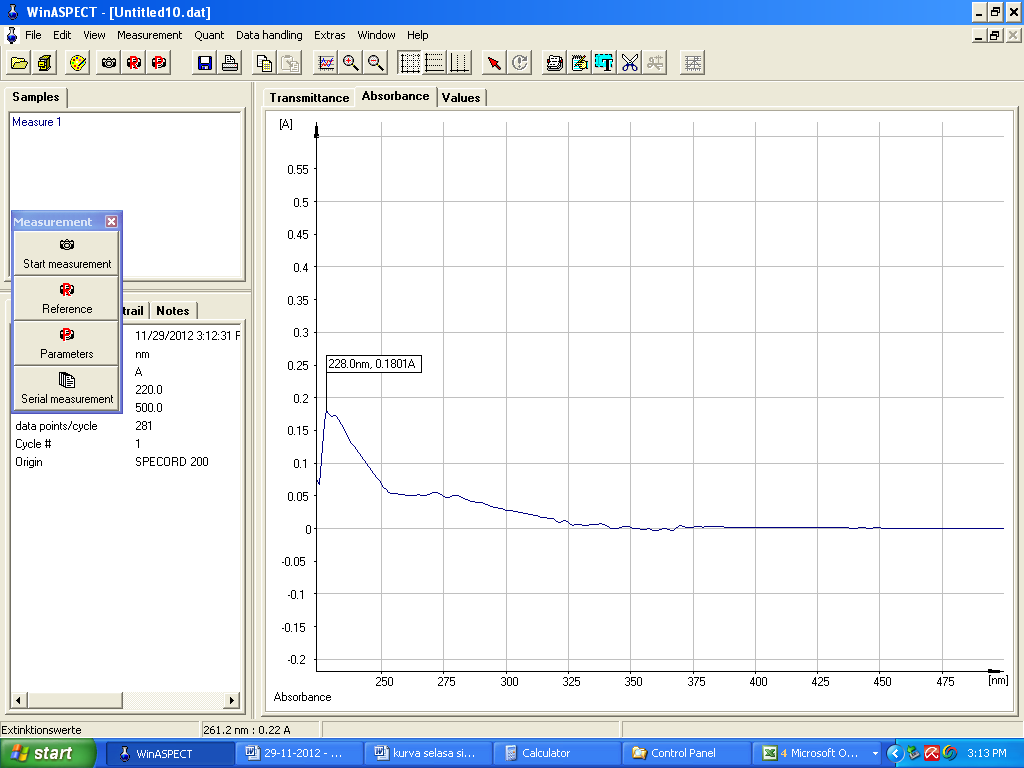


UV spectrum of GC1 compound that isolated from *G. celebica* leaves

**GC 1: continued**

**
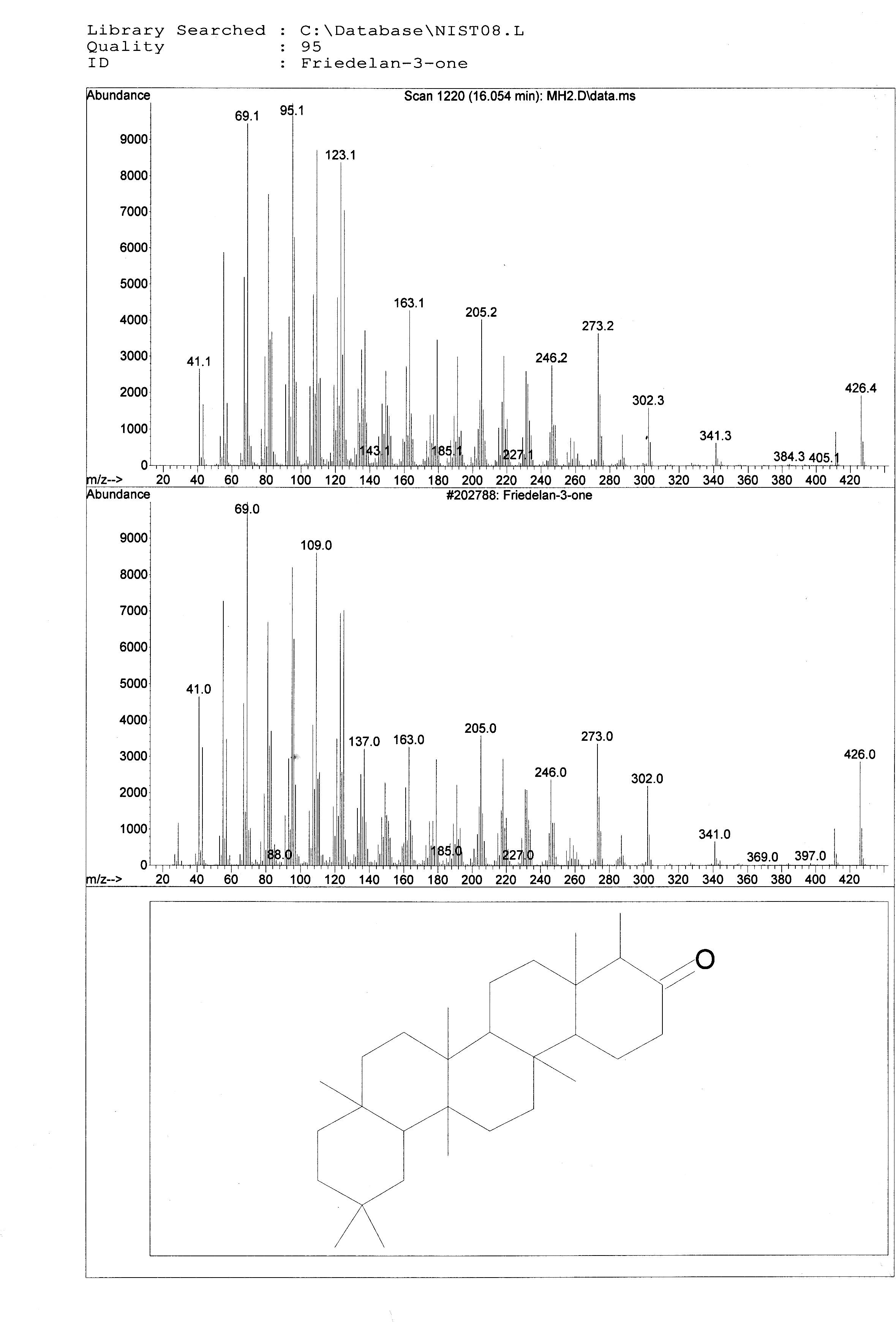
**

Mass Spectrum Using GCMS in MeOH-CHCl_3_ of GC1 compound that isolated from *G. celebica* Leaves


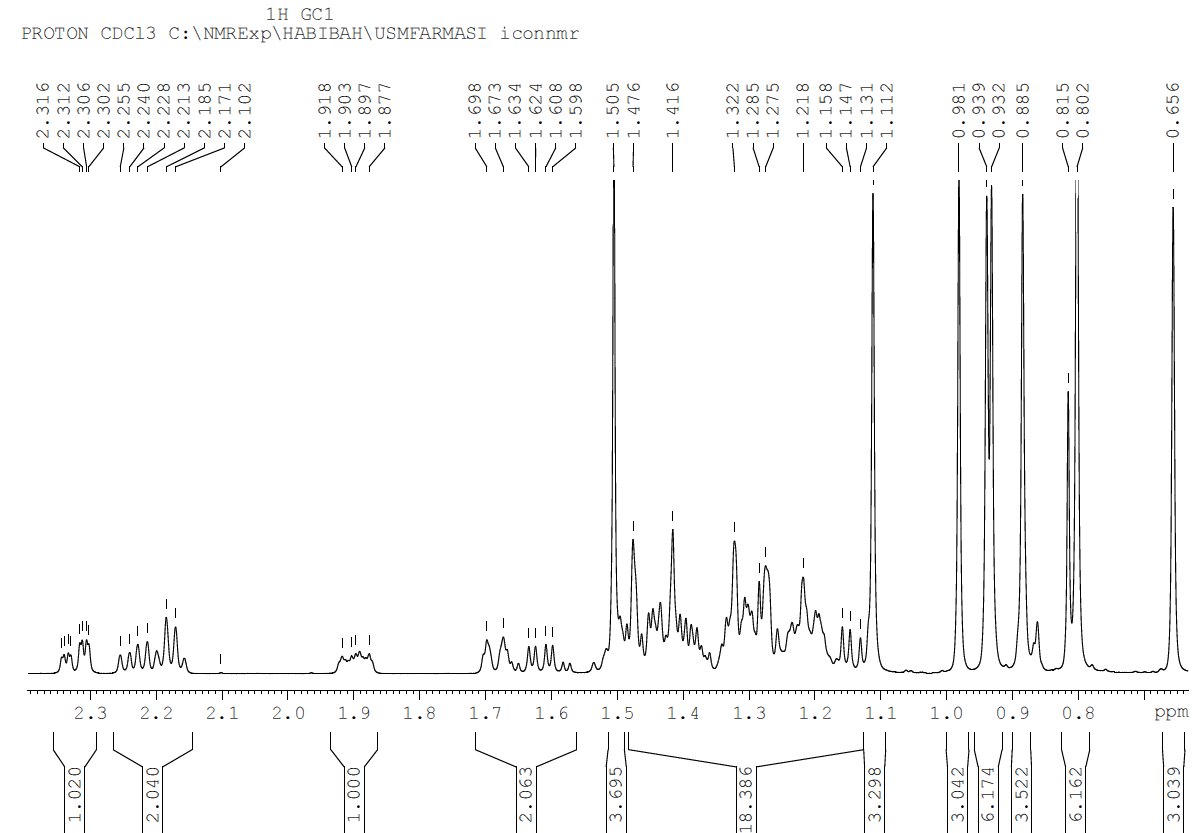


1H-NMR spectrum of GC1 compound that isolated from *G. celebica* leaves using CDCl_3_ solvent

**GC1: continued**

^
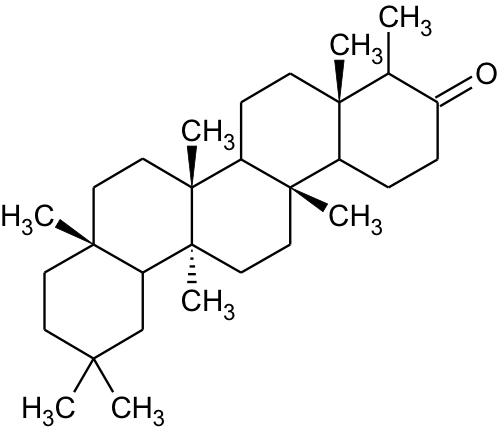

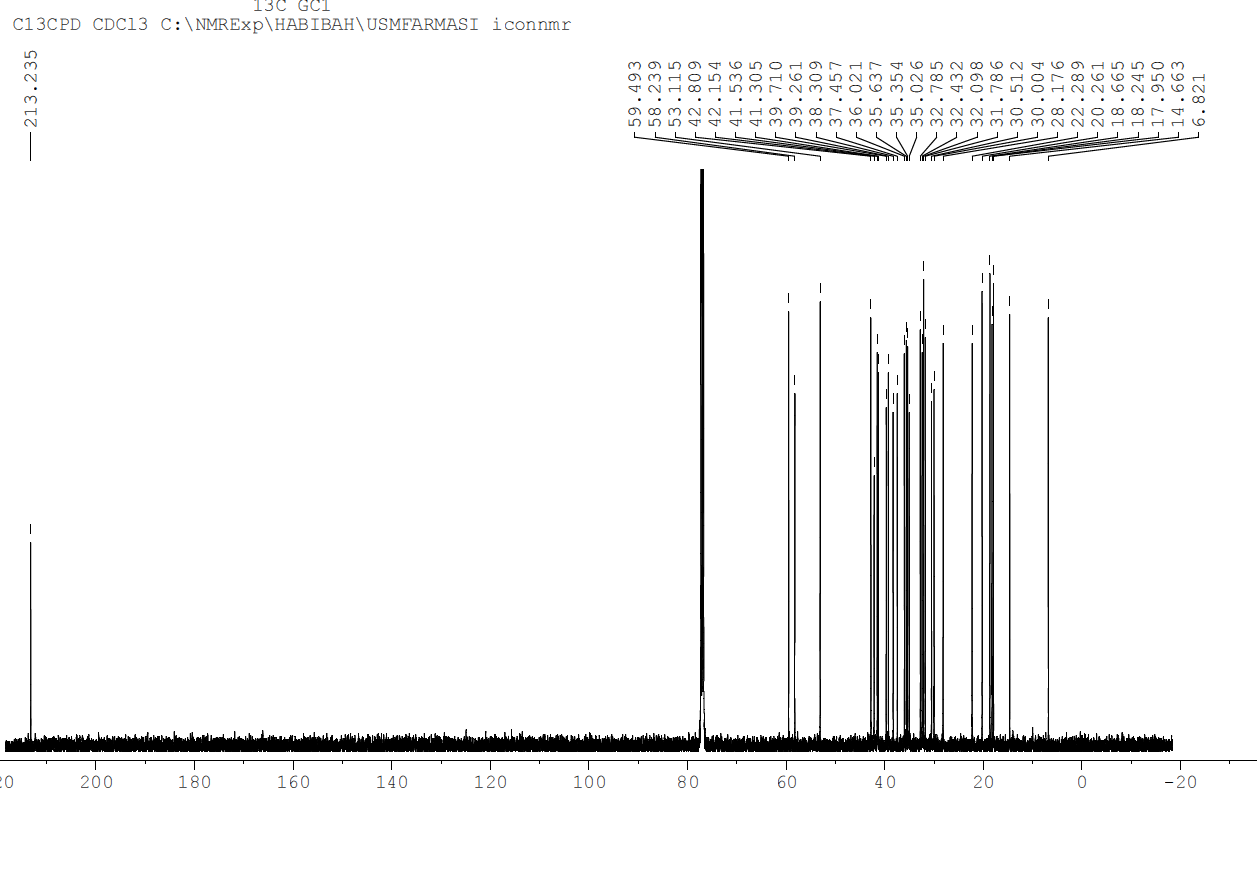
^

^
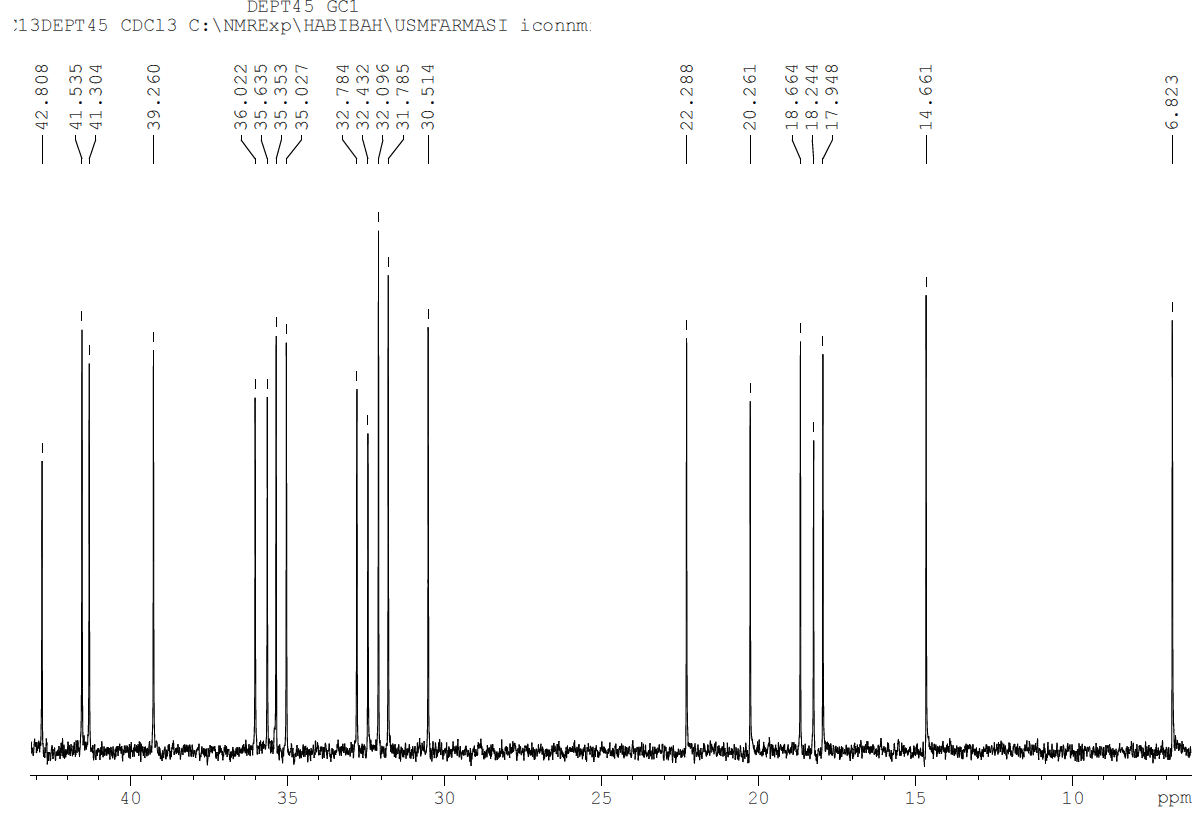
^

^13^C and DEPT45-NMR (top to bottom) spectrum of GC1 compound that isolated from *G. celebica* leaves using CDCl_3_ solvent

**GC 1: continued**


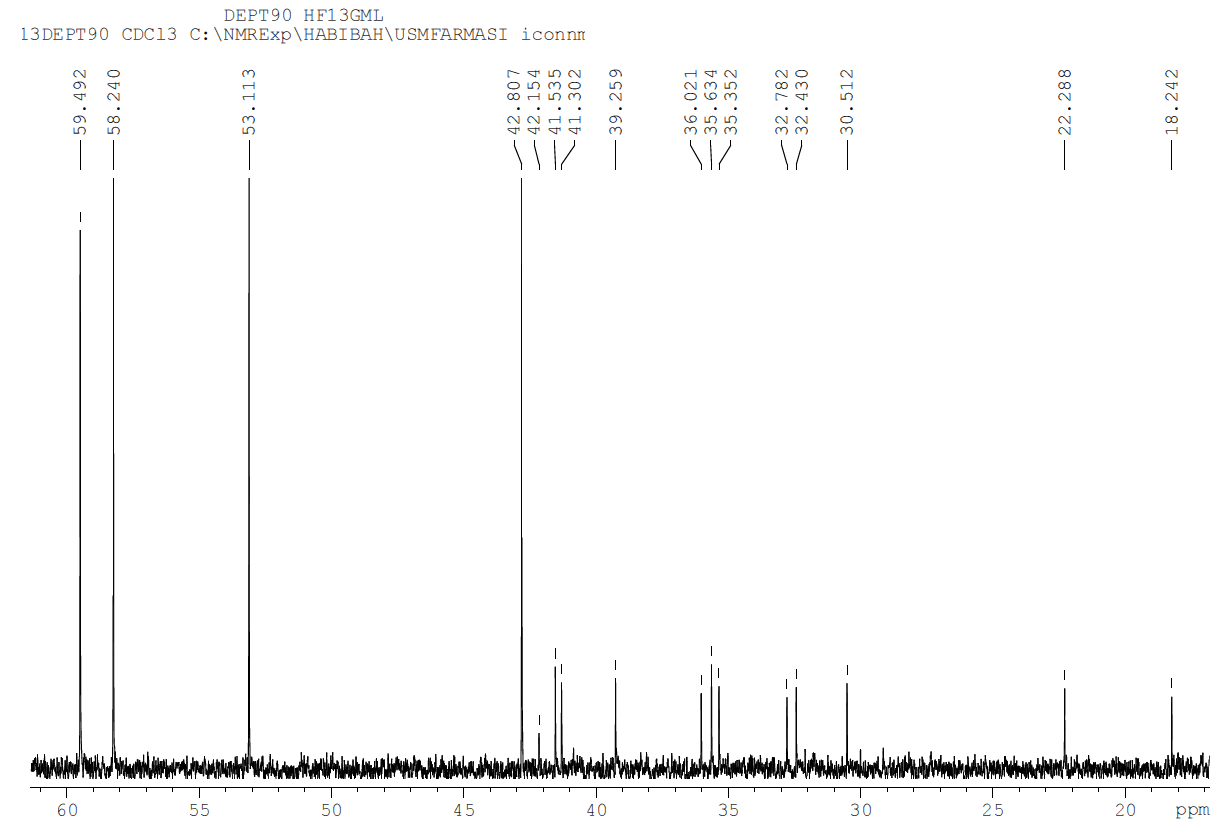


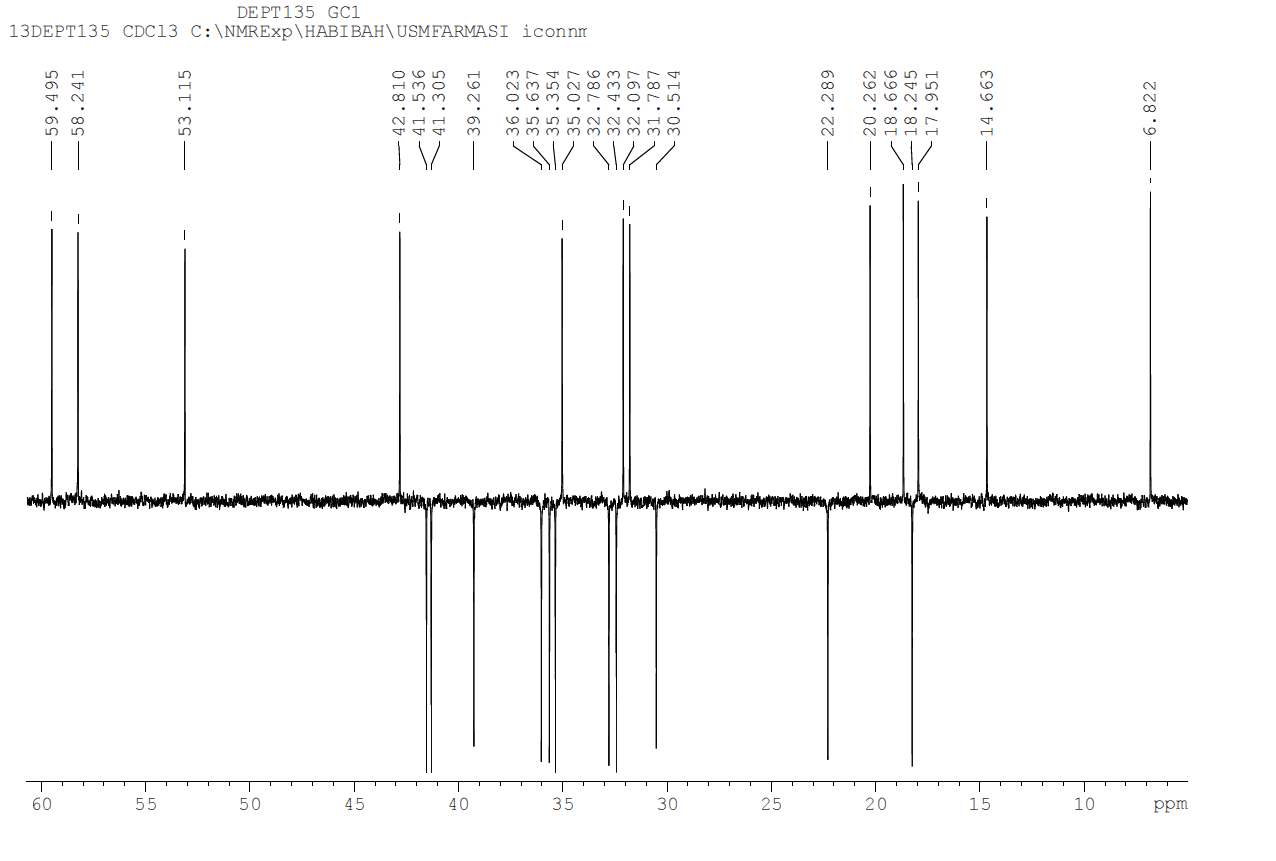


DEPT90 and DEPT135-NMR (top to bottom) spectrum of GC1 compound that isolated from *G. celebica* leaves using CDCl_3_ solvent

**GC 1: continued**

**
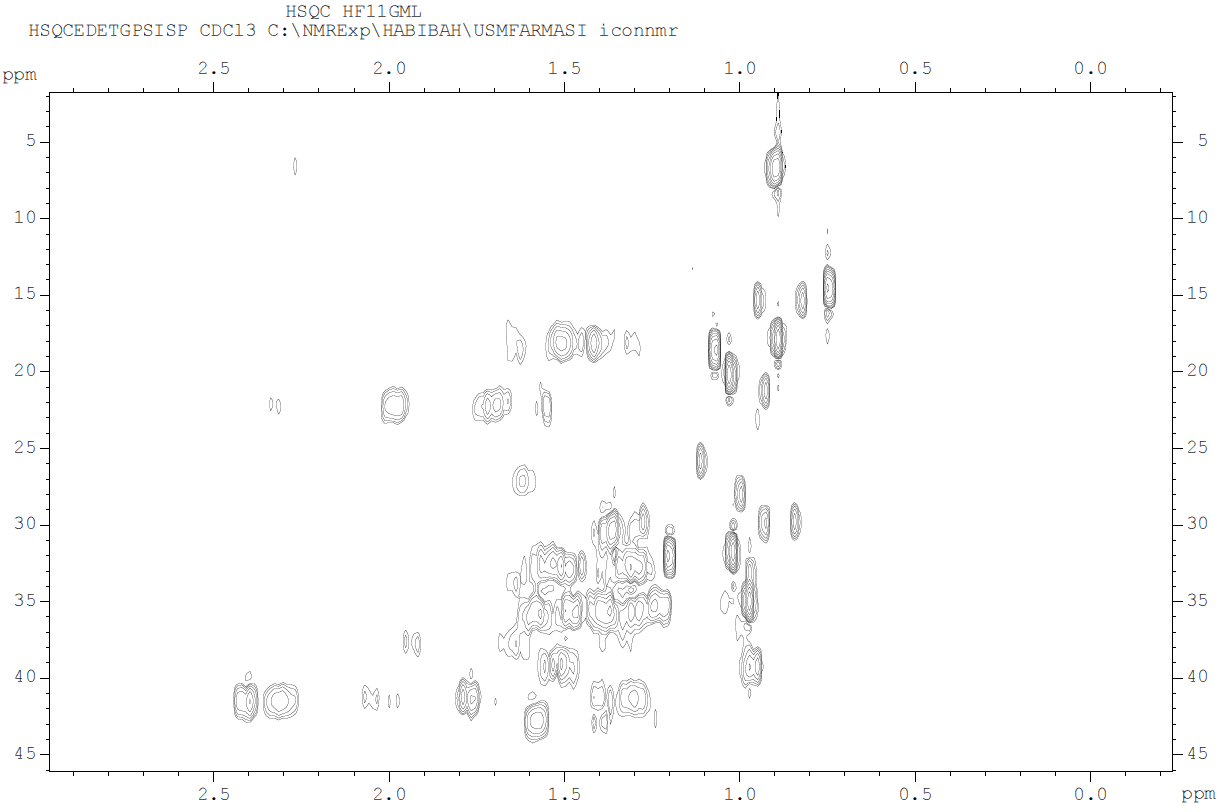
**

2D-HSQC-NMR of **GC1** using CDCl_3_ solvent


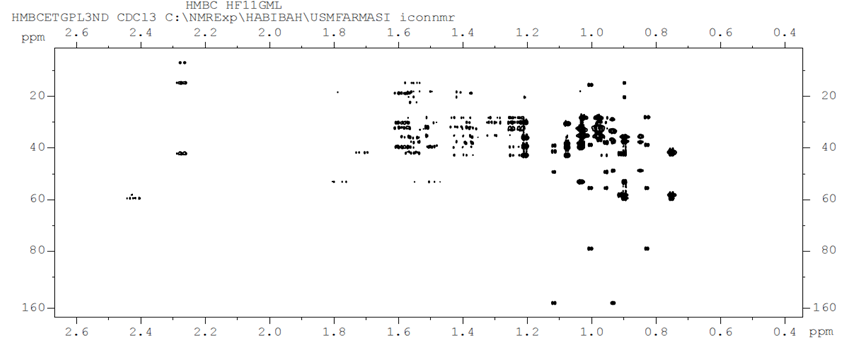


2D-HMBC-NMR of GC1 using CDCl_3_ solvent

**GC 1: continued**


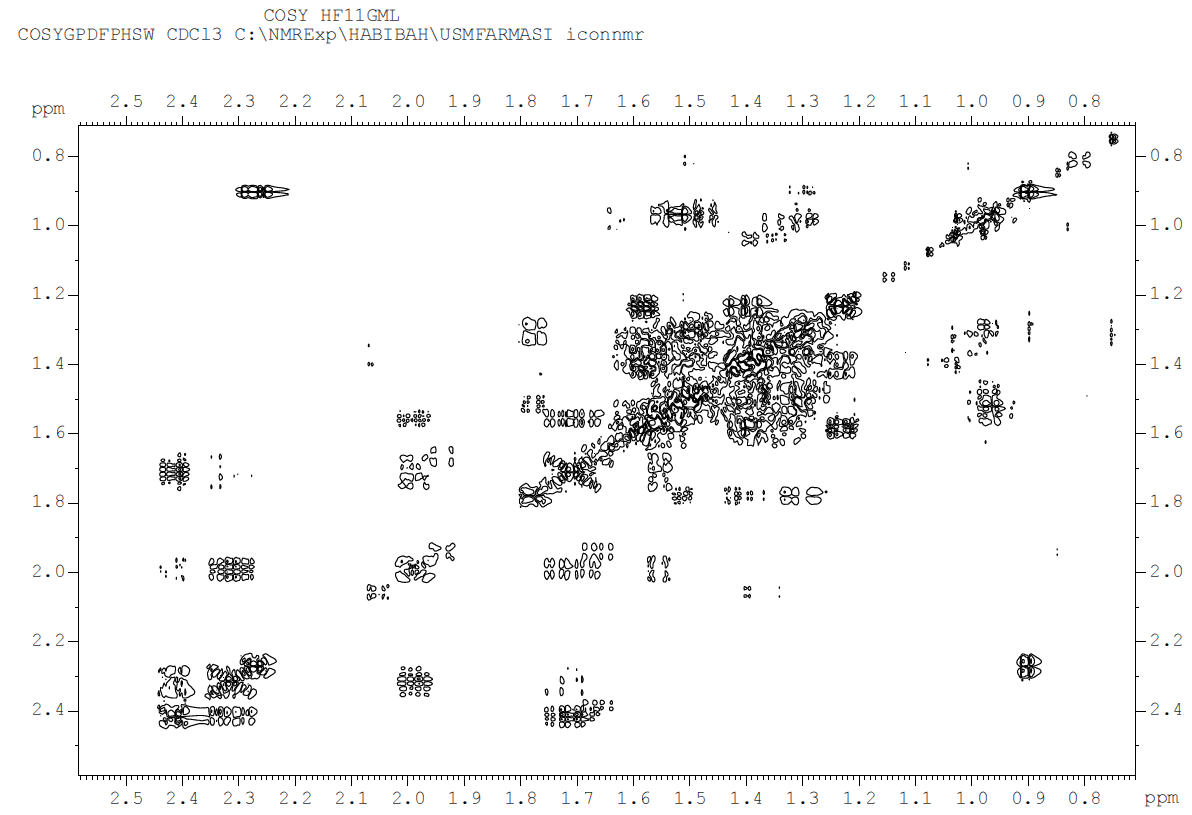


2D-COSY-NMR of GC1 using CDCl_3_ solvent

**Spectroscopy data of GC2**

IR spectrum of GC2 compound that isolated from *G. celebica* leaves


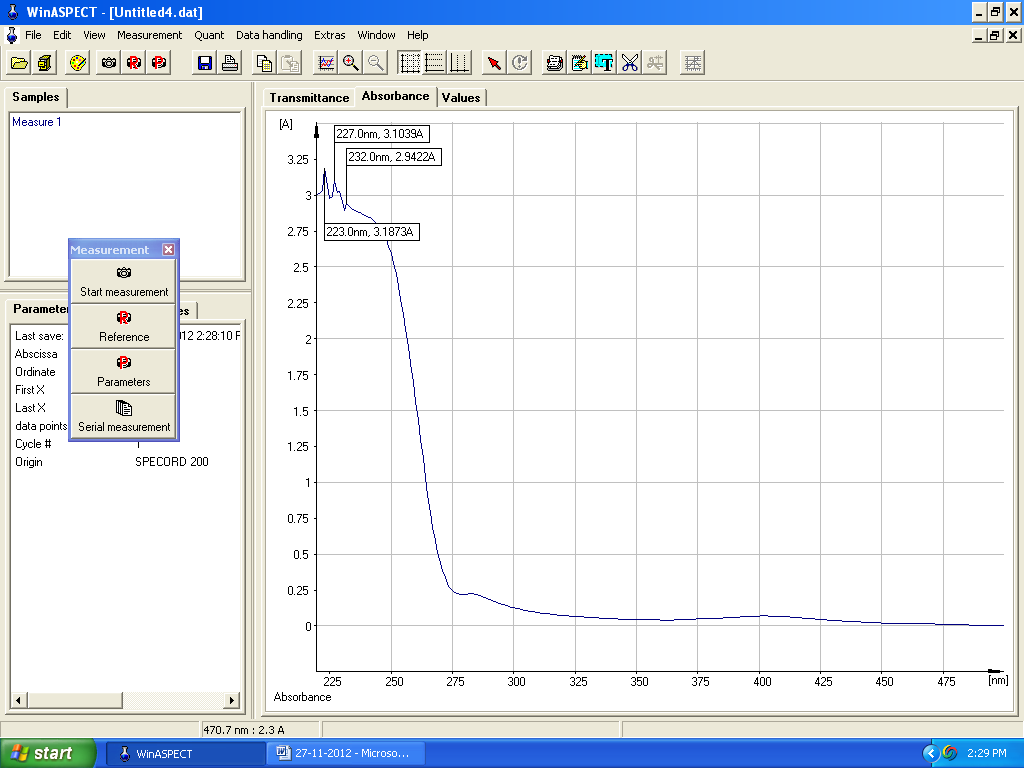


UV spectrum of GC2 compound that isolated from *G. celebica* leaves

**GC2: continued**

**
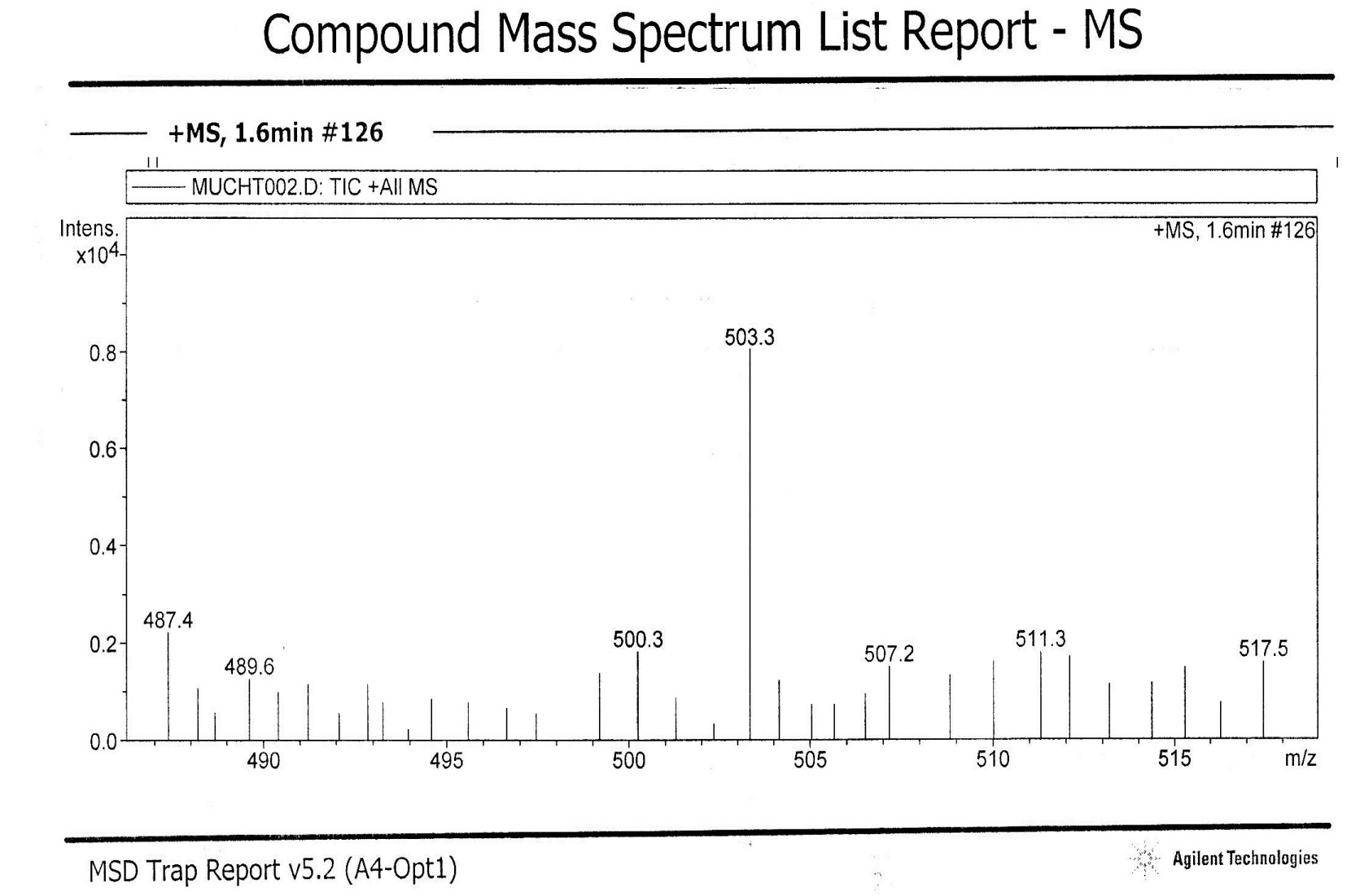
**

**
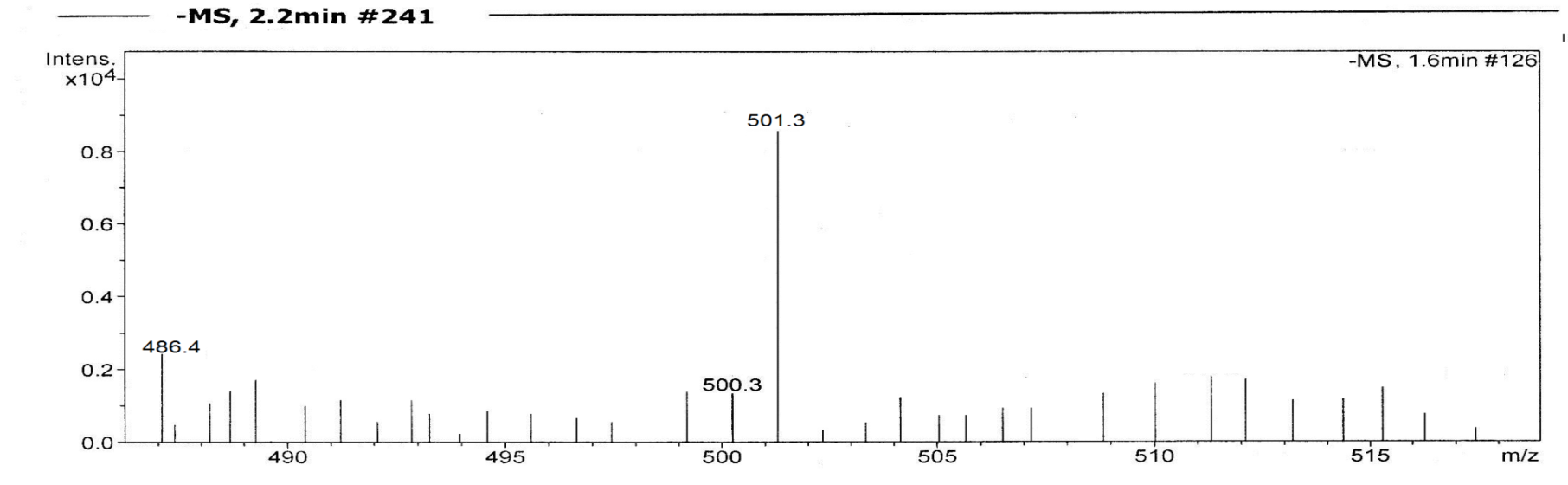
**

Positive Mode [M+H]^+^ (bottom) of Mass Spectrum Using ESIMS-Trap-Direct Injection in MeOH of GC2 compound that isolated from *G. celebica* Leaves

**GC2: continued**


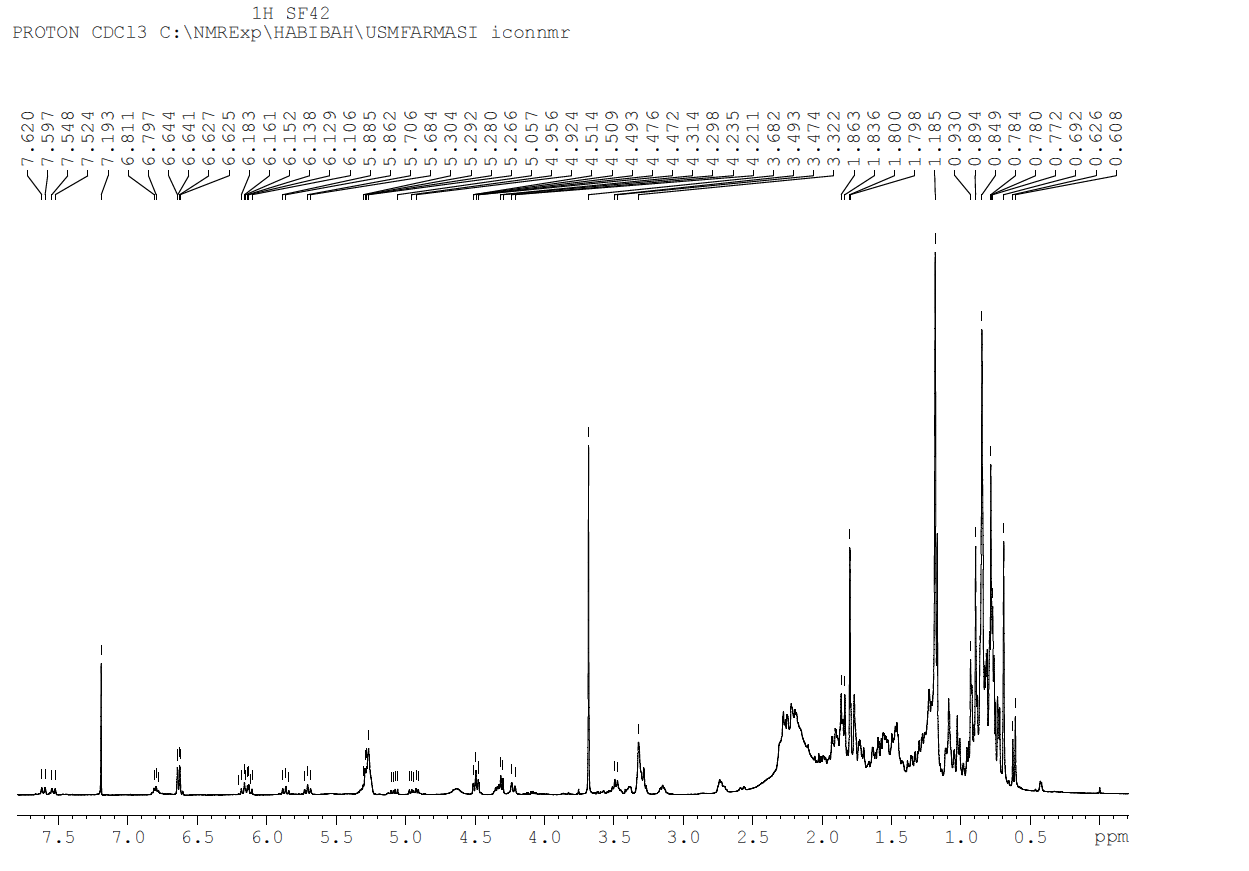


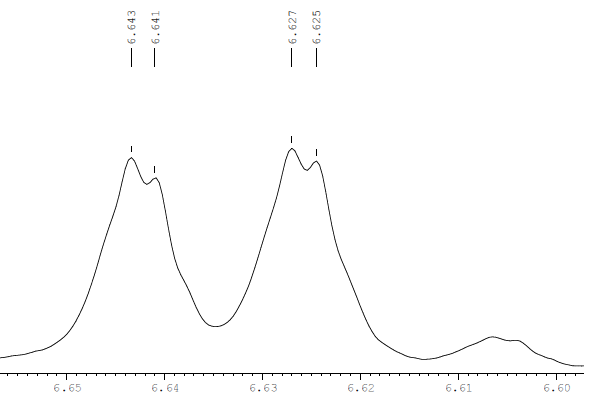

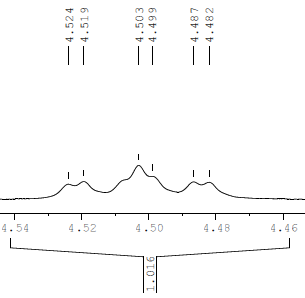

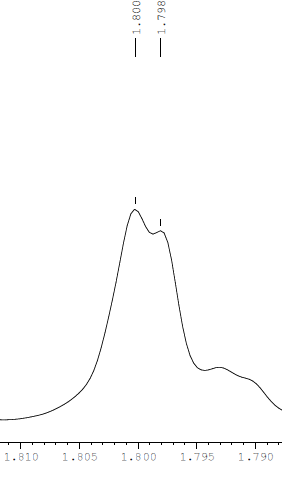

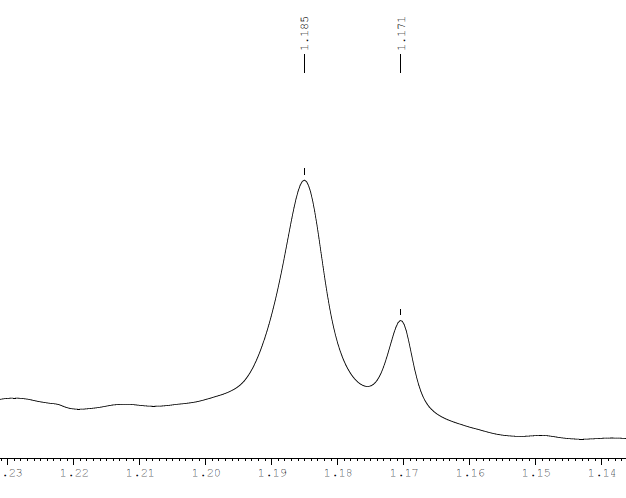


1H-NMR spectrum of GC2 compound that isolated from *G. celebica* Leaves using CDCl_3_ solvent

**GC2: continued**

^
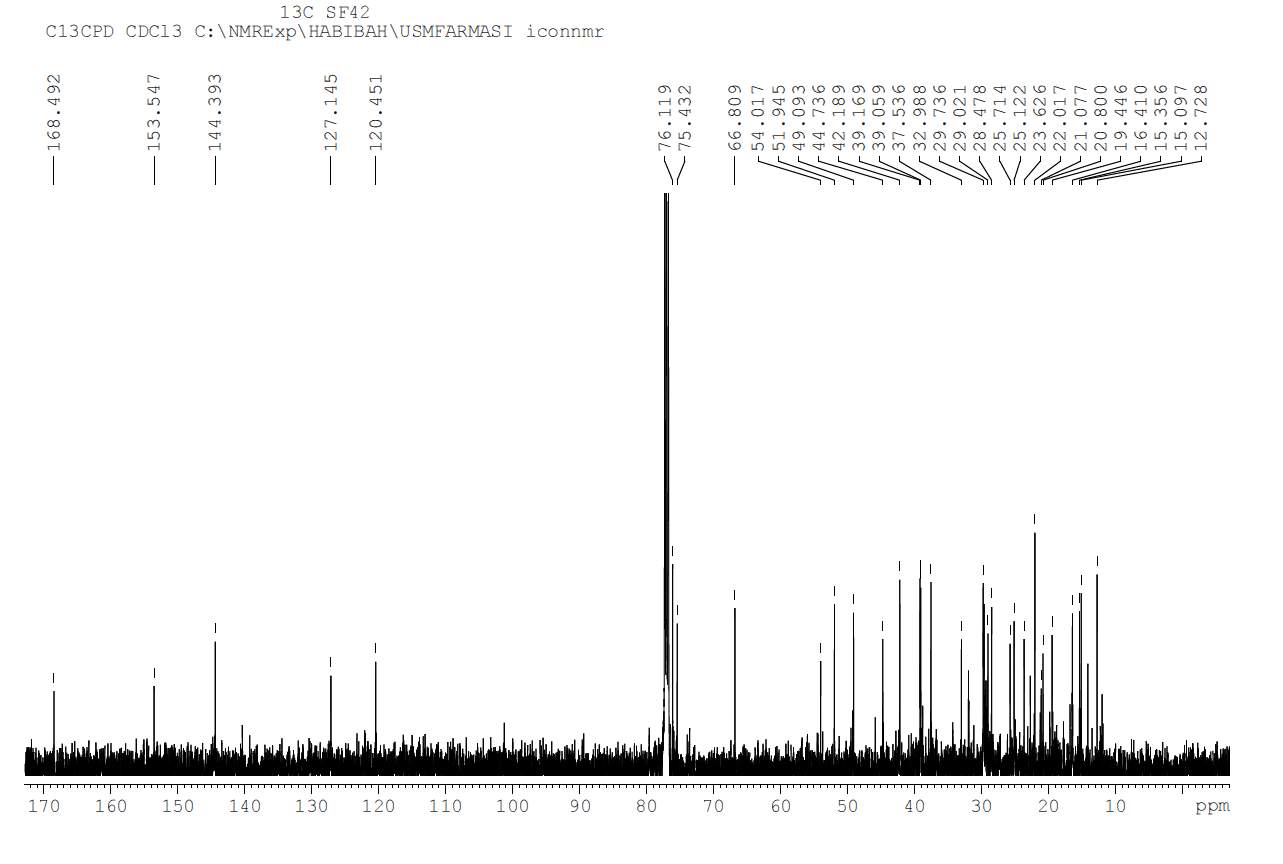
^

^
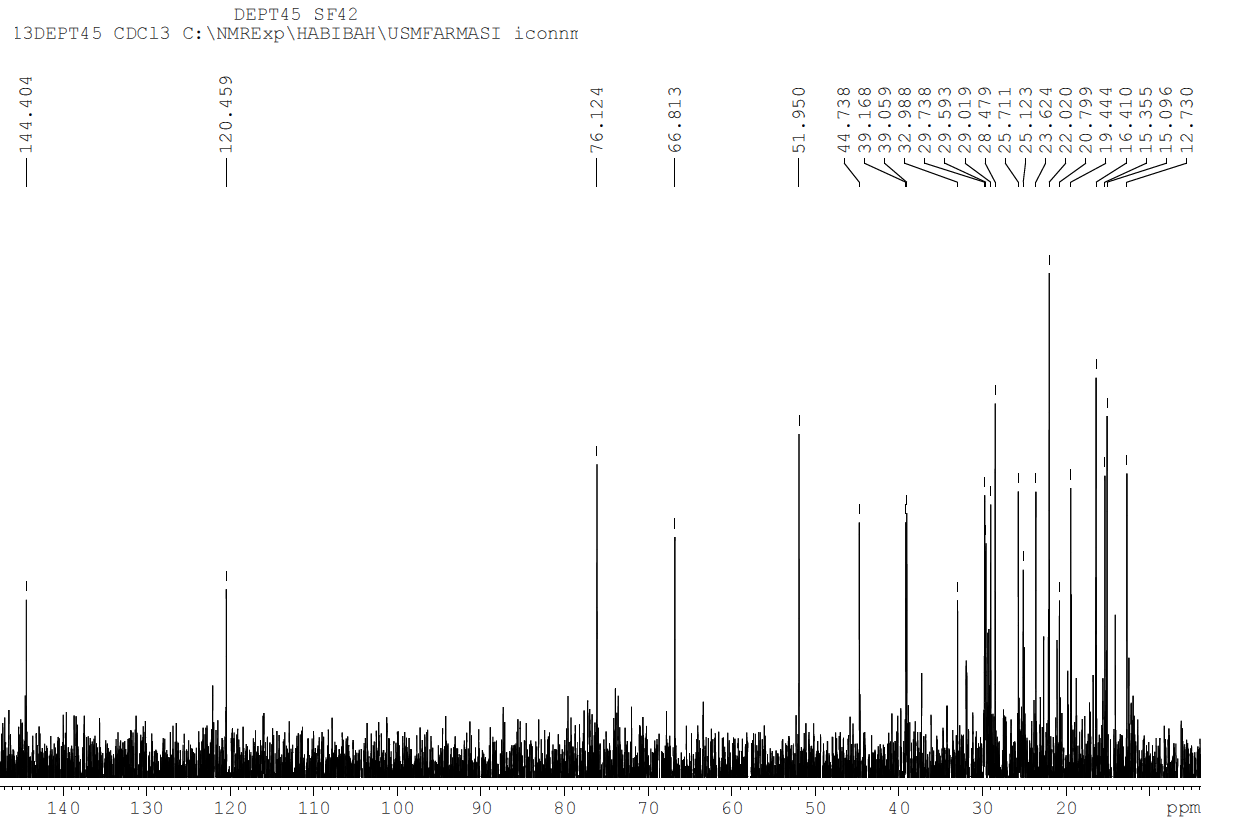
^

^13^C and DEPT45-NMR (top to bottom) spectrum of GC2 compound that isolated from *G. celebica* Leaves using CDCl_3_ solvent

**GC2: continued**


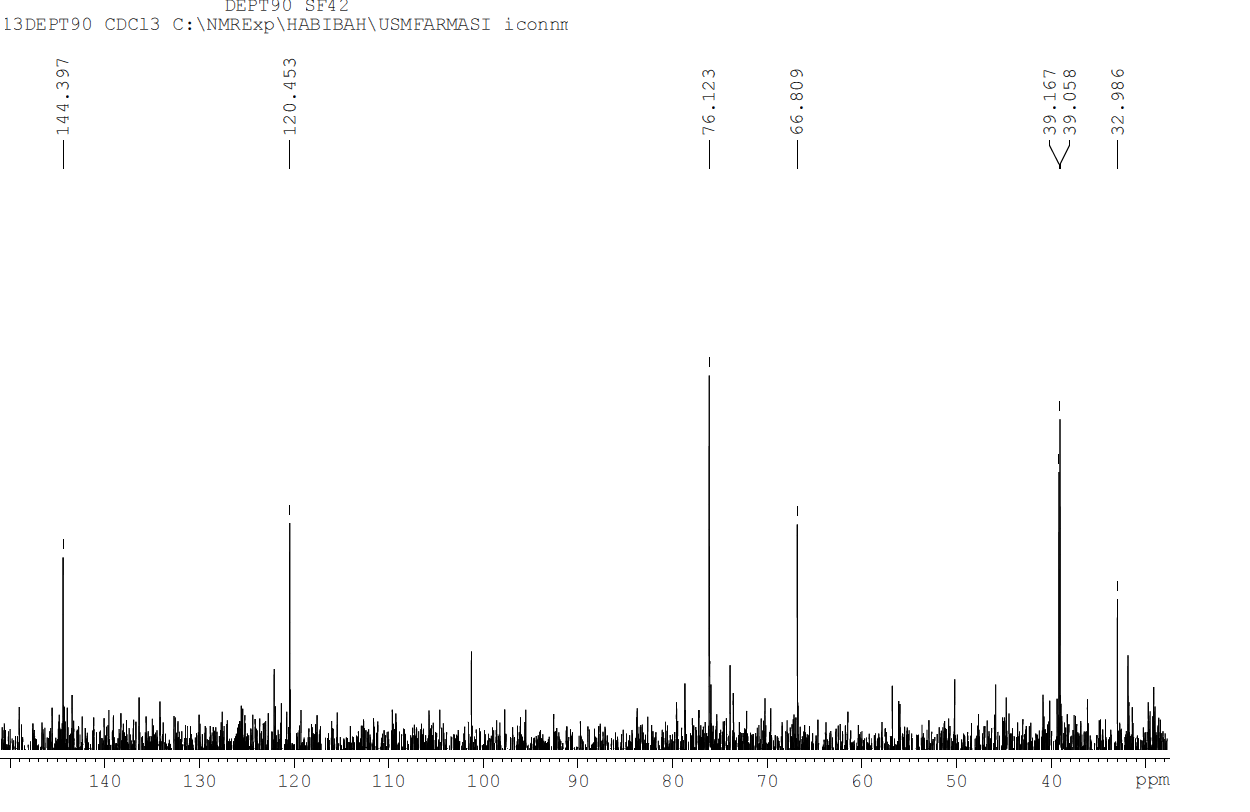


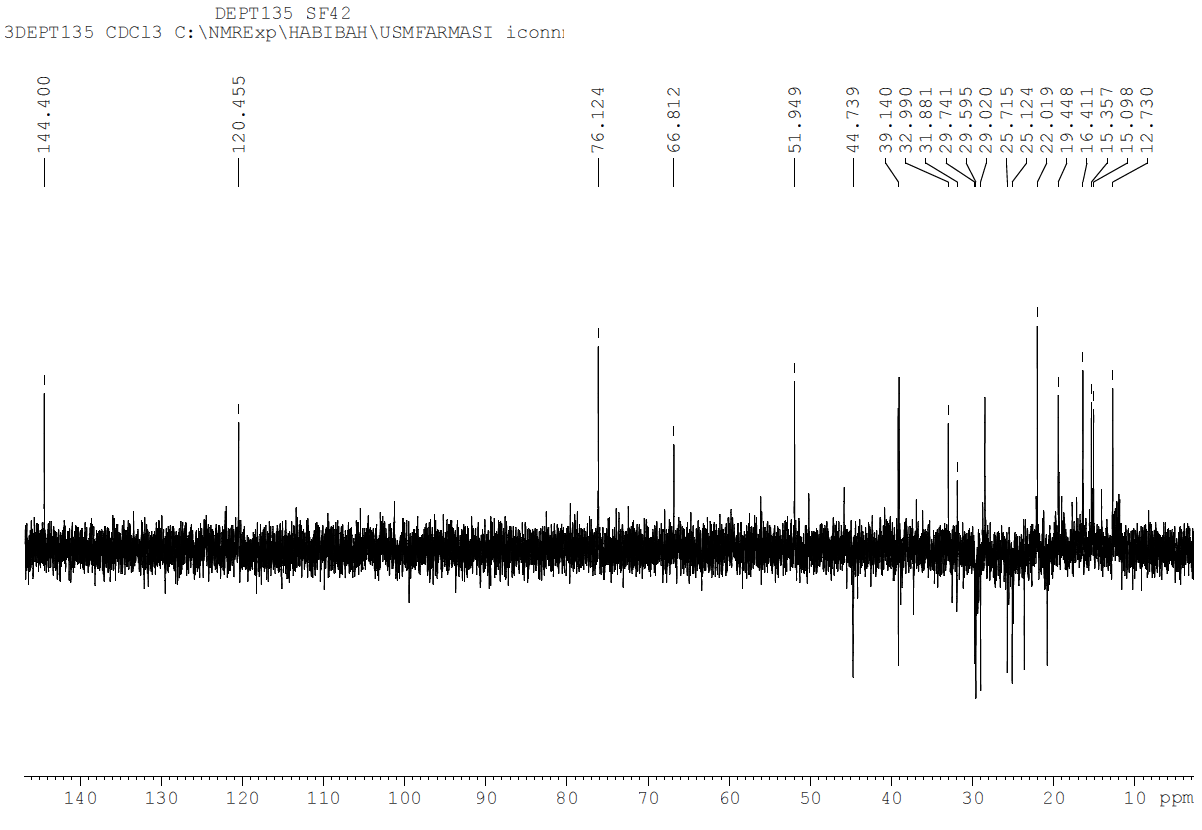


DEPT90 and DEPT135-NMR (top to bottom) spectrum of GC2 compound that isolated from *G. celebica* leaves using CDCl_3_ solvent

**GC2: continued**

**
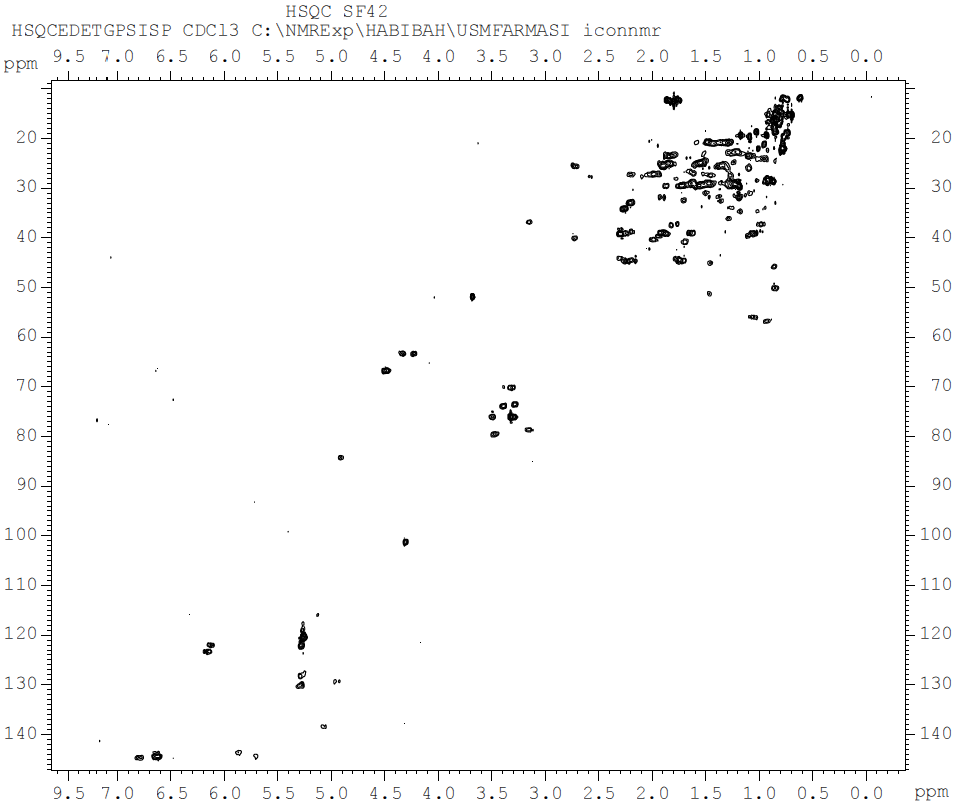
**

**
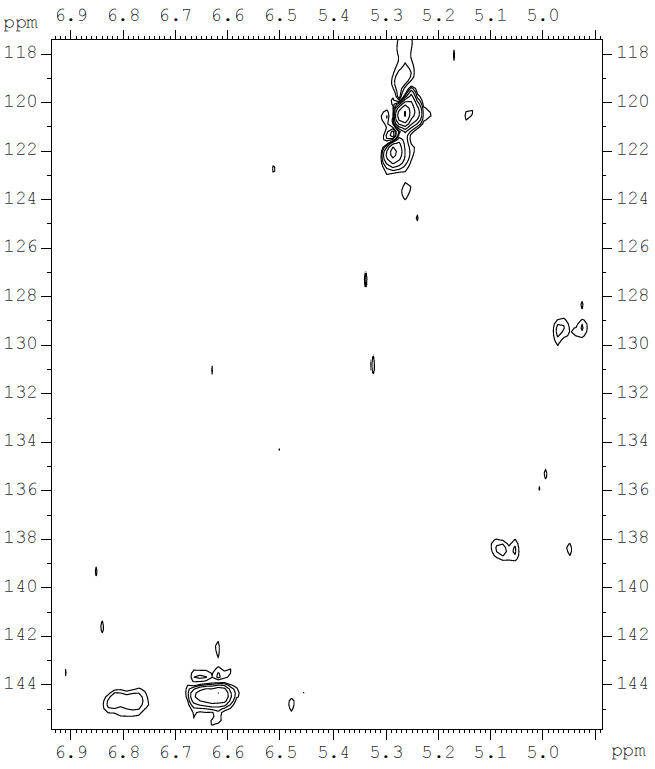

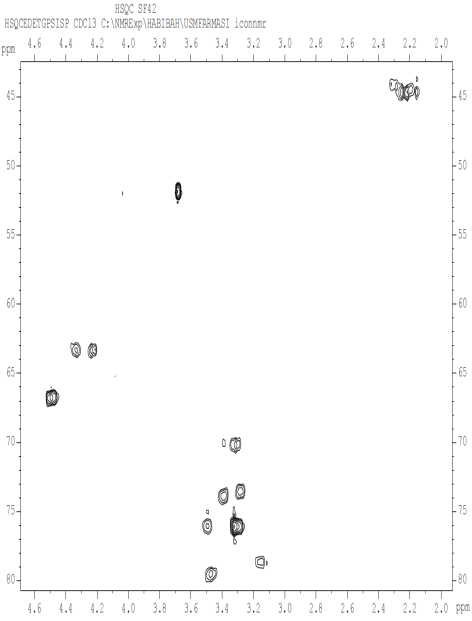

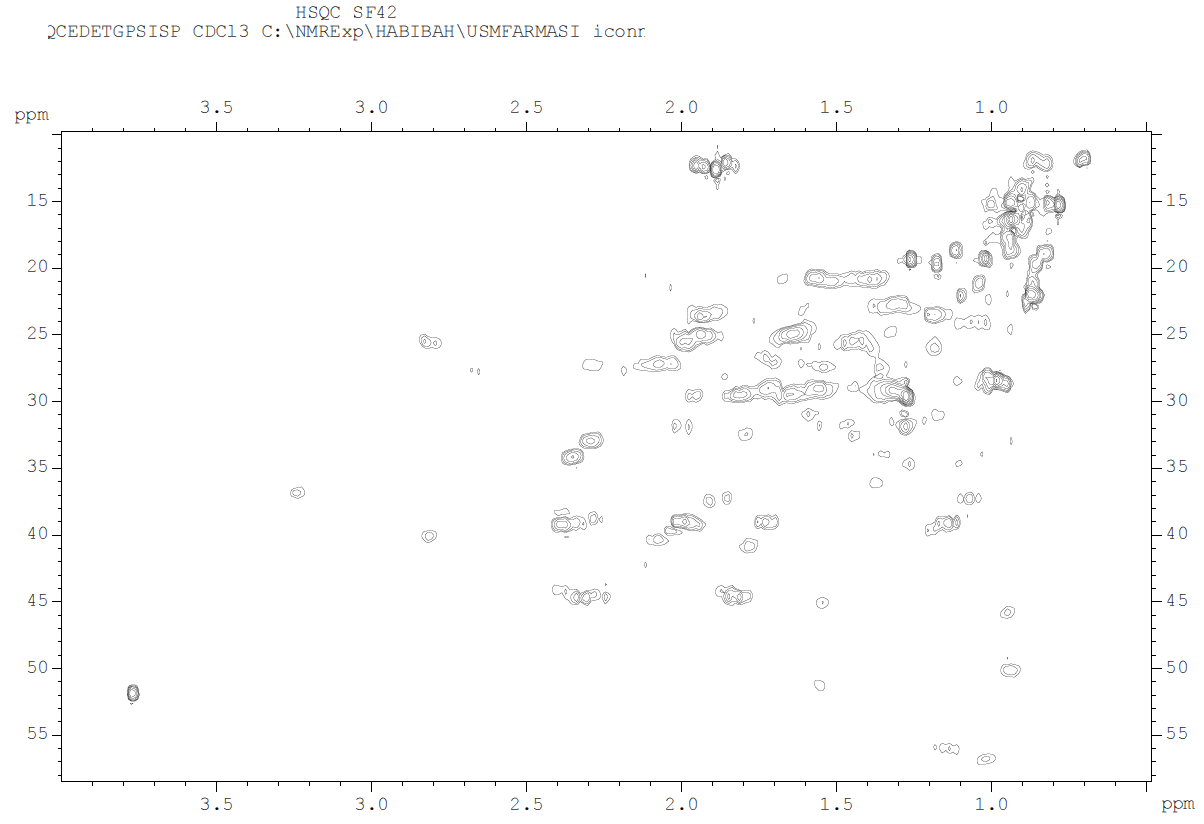
**

2D-HSQC-NMR of GC2 using CDCl_3_ solvent

**GC2: continued**


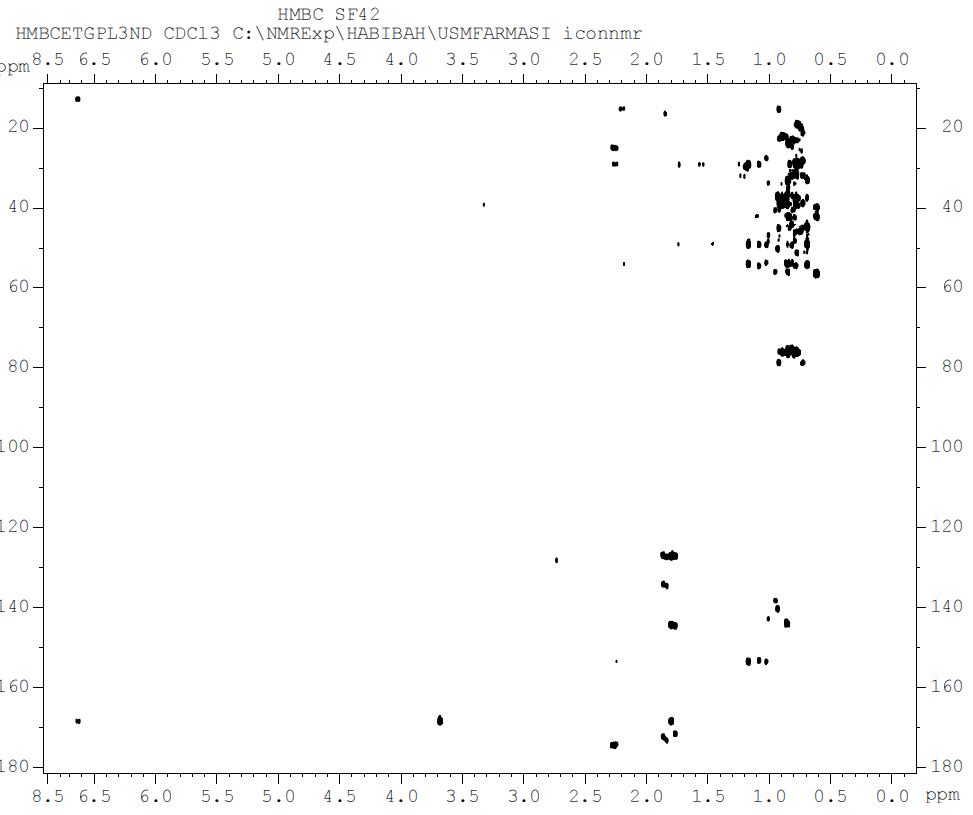


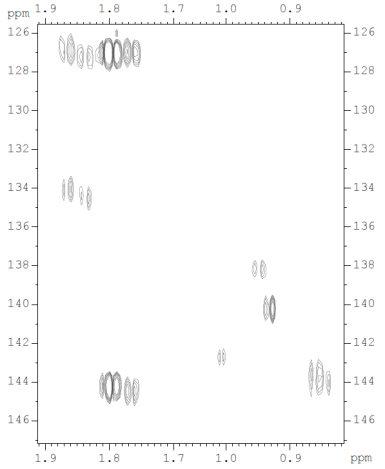

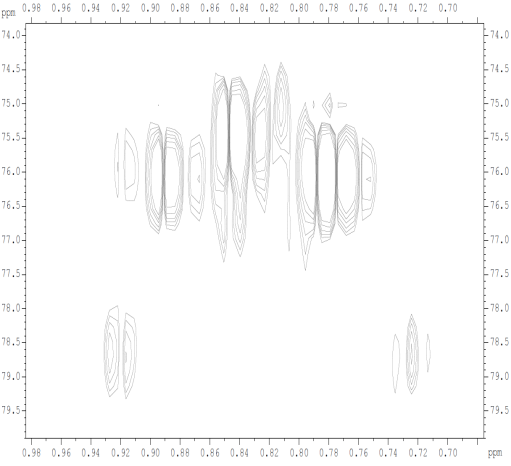

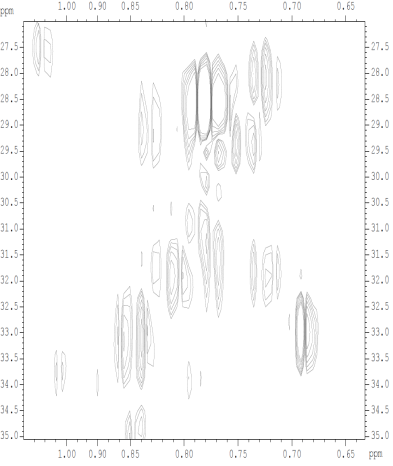

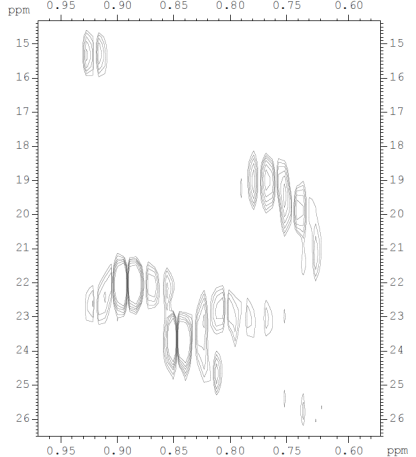


2D-HMBC-NMR of GC2 using CDCl_3_ solvent

**GC2: continued**


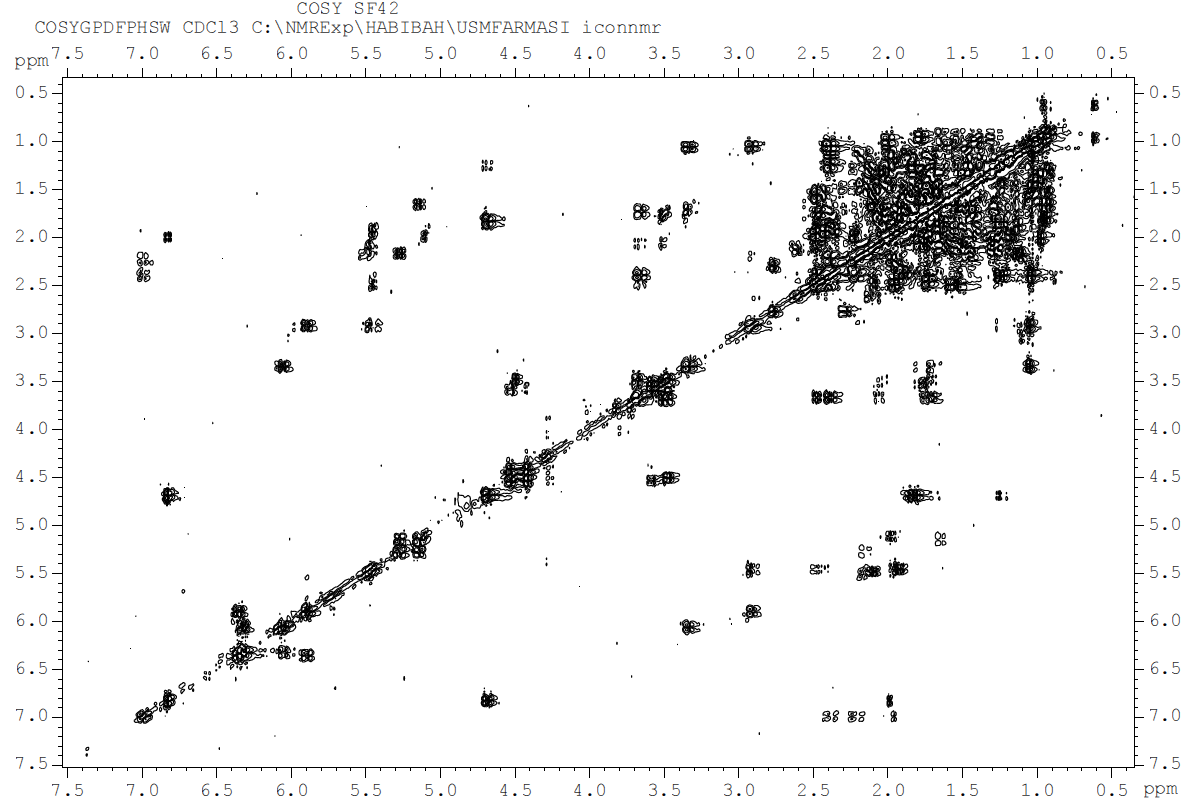


2D-COSY-NMR of GC2 using CDCl_3_ solvent

**Spectroscopy data of GC3**


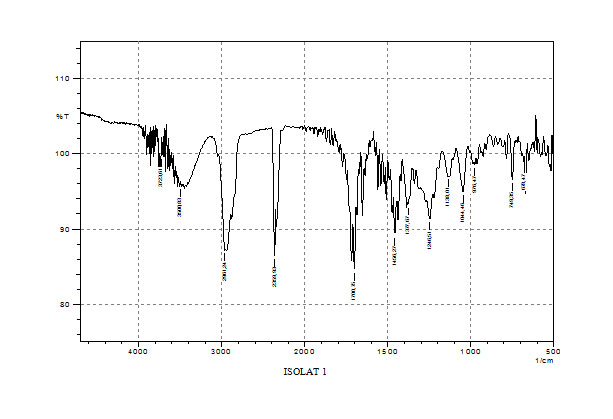


IR spectrum of GC3 compound that isolated from *G. celebica* leaves

UV spectrum of GC3 compound that isolated from *G. celebica* leaves

**GC3: continued**

**
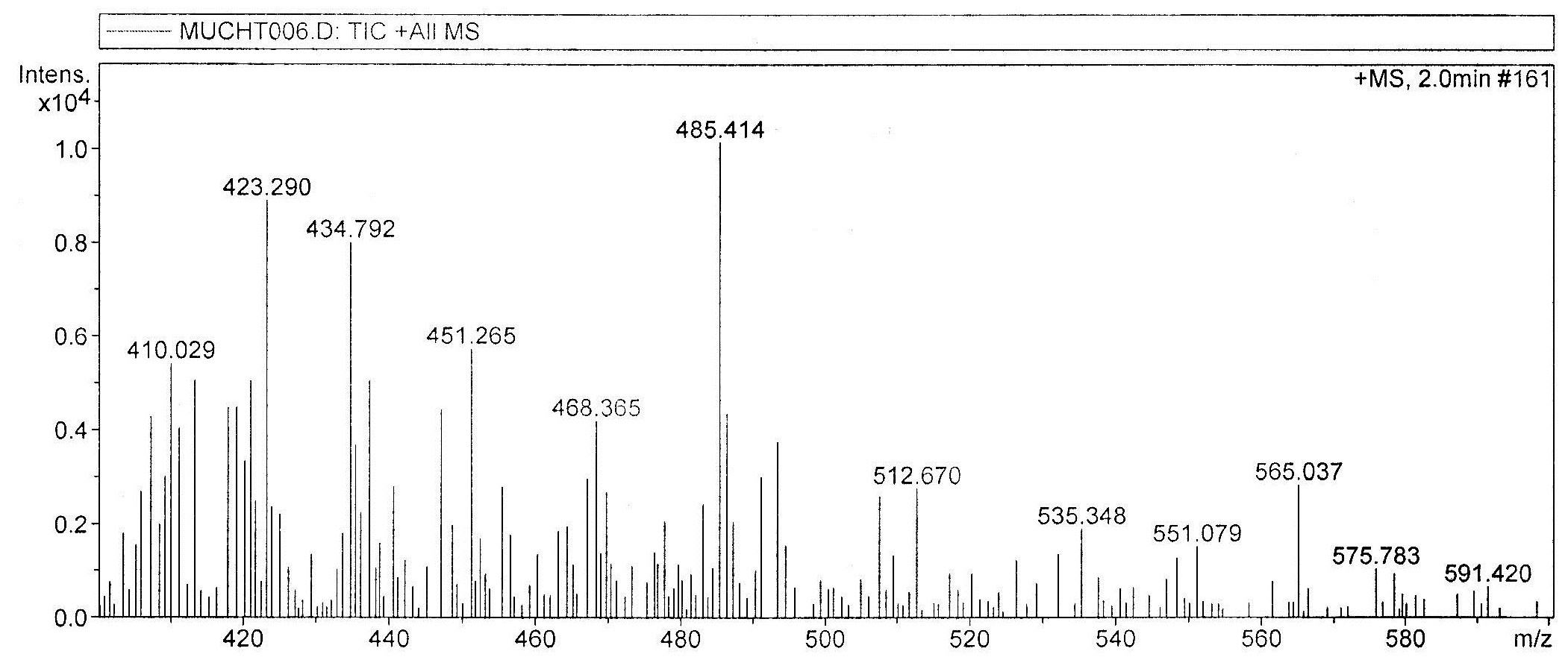
**

**
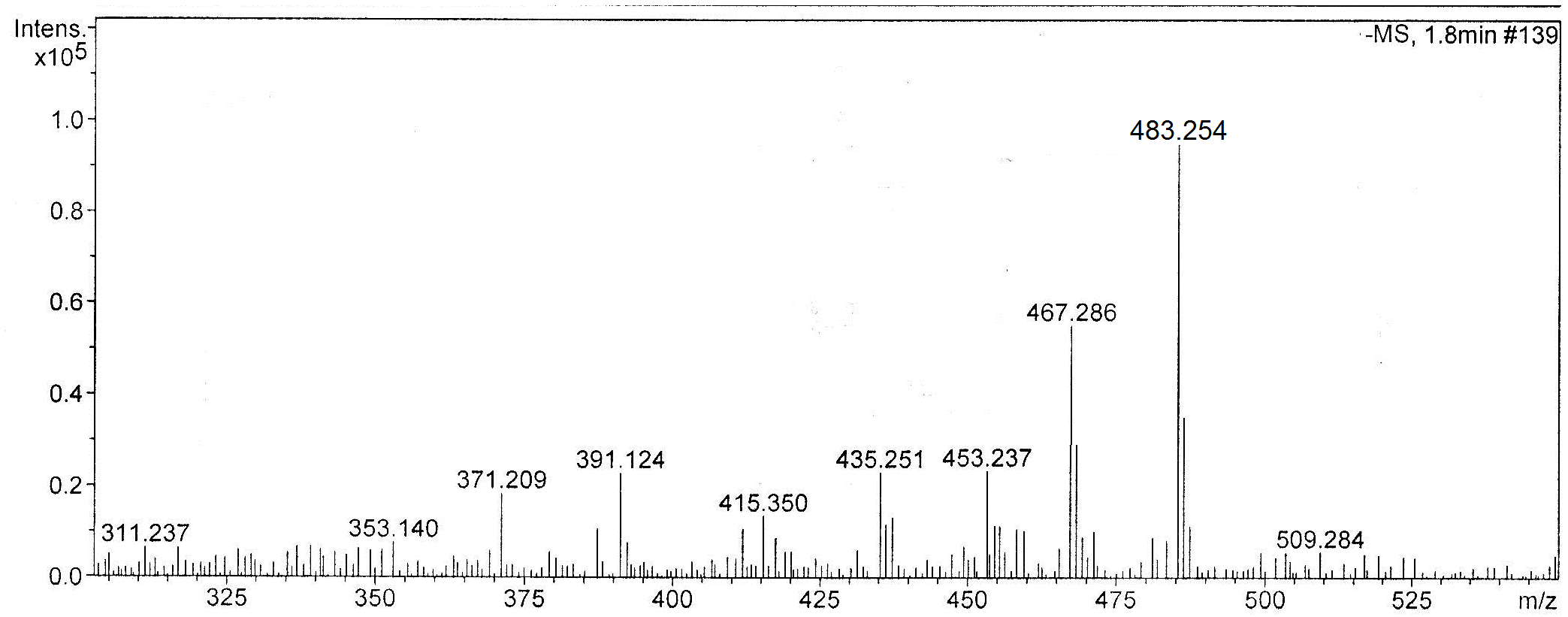
**

Positive Mode [M+H]^+^ and Negative Mode [M-H]^+^  (top to bottom) of Mass Spectrum Using ESIMS-Trap-Direct Injection in MeOH of GC3 compound that isolated from *G. celebica* Leaves

**GC3: continued**


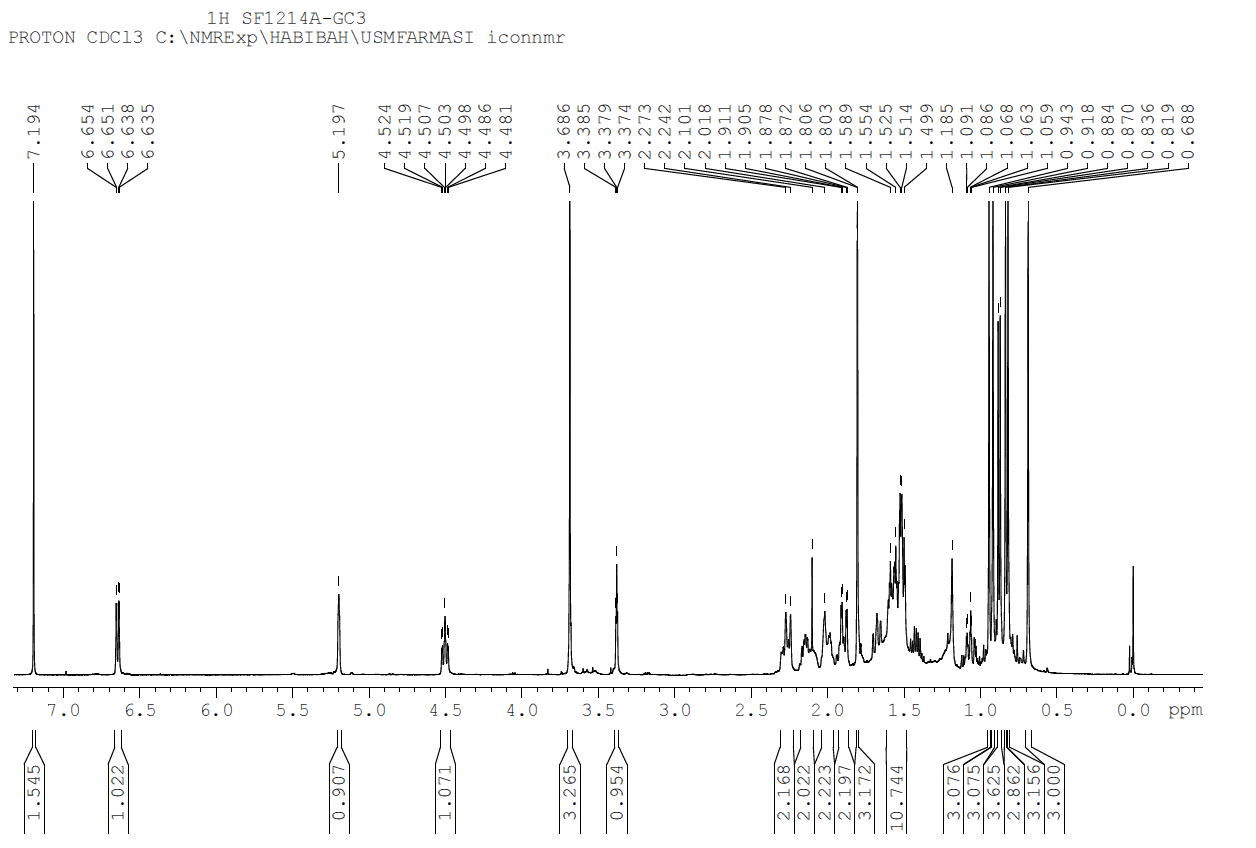


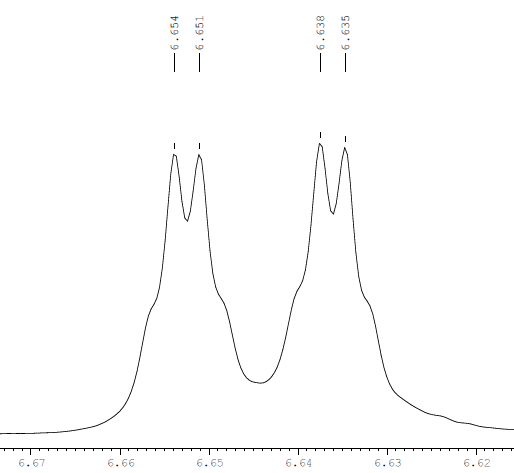

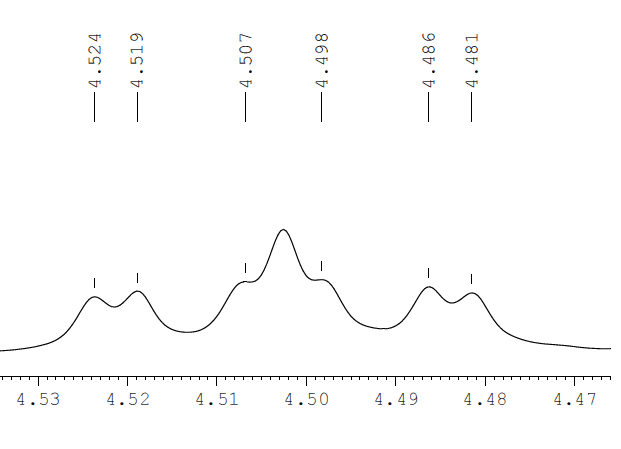

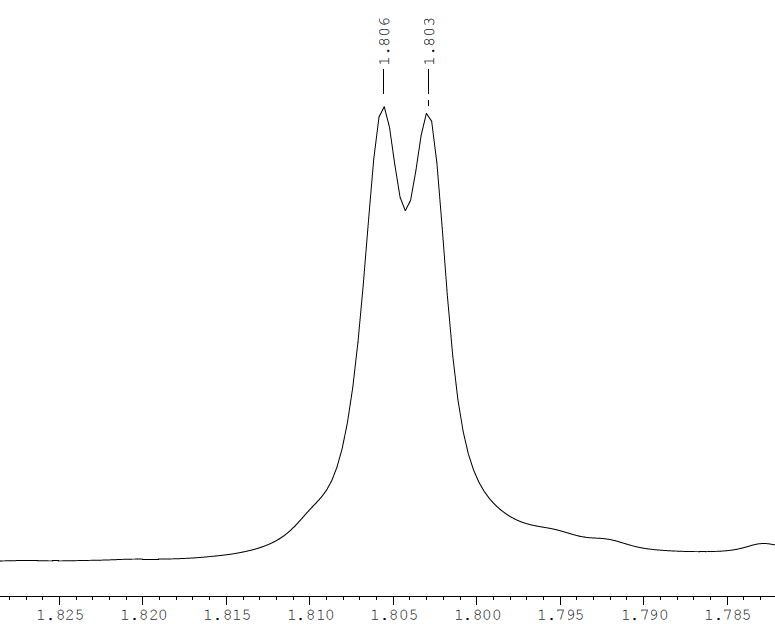

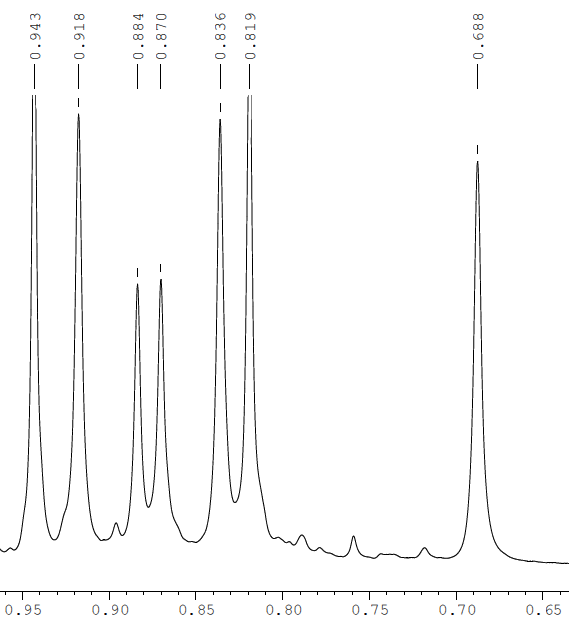


1H-NMR spectrum of GC3 compound that isolated from *G. celebica* Leaves using CDCl_3_ solvent

**GC3: continued**

^
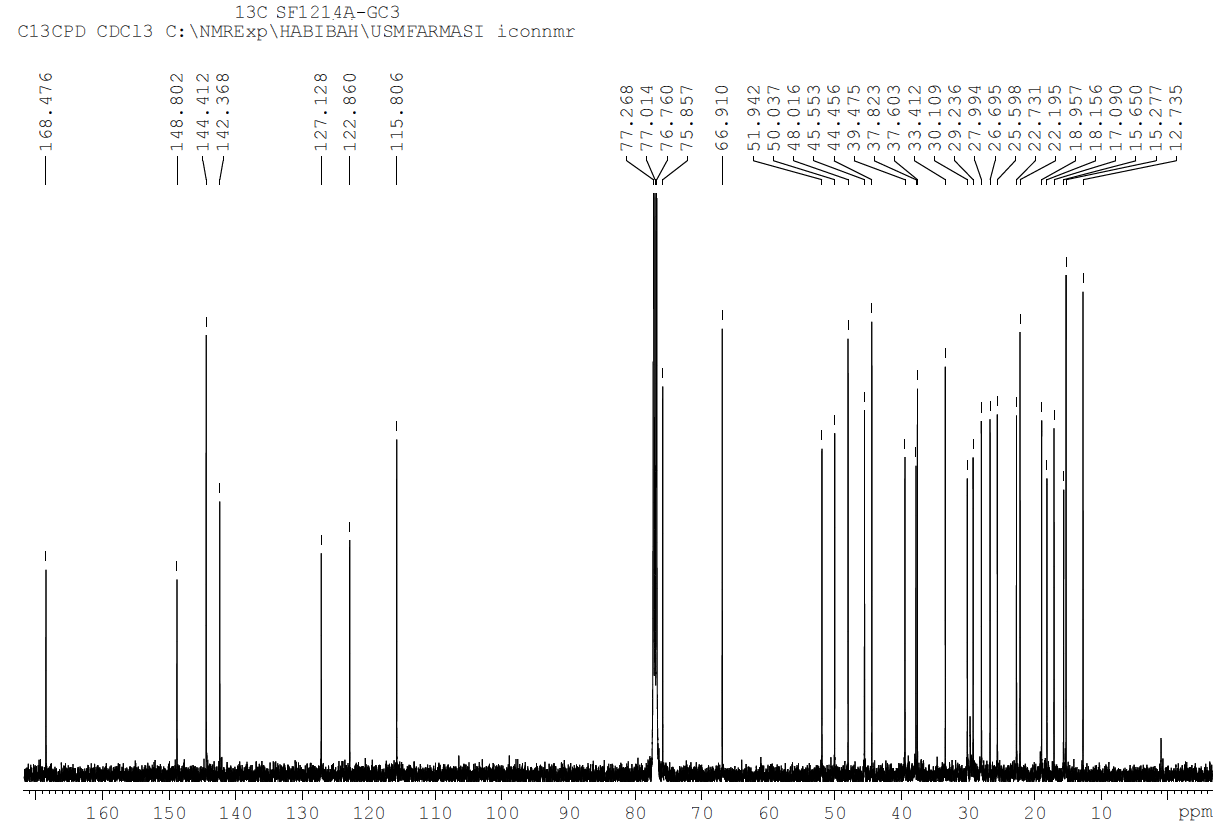
^

^
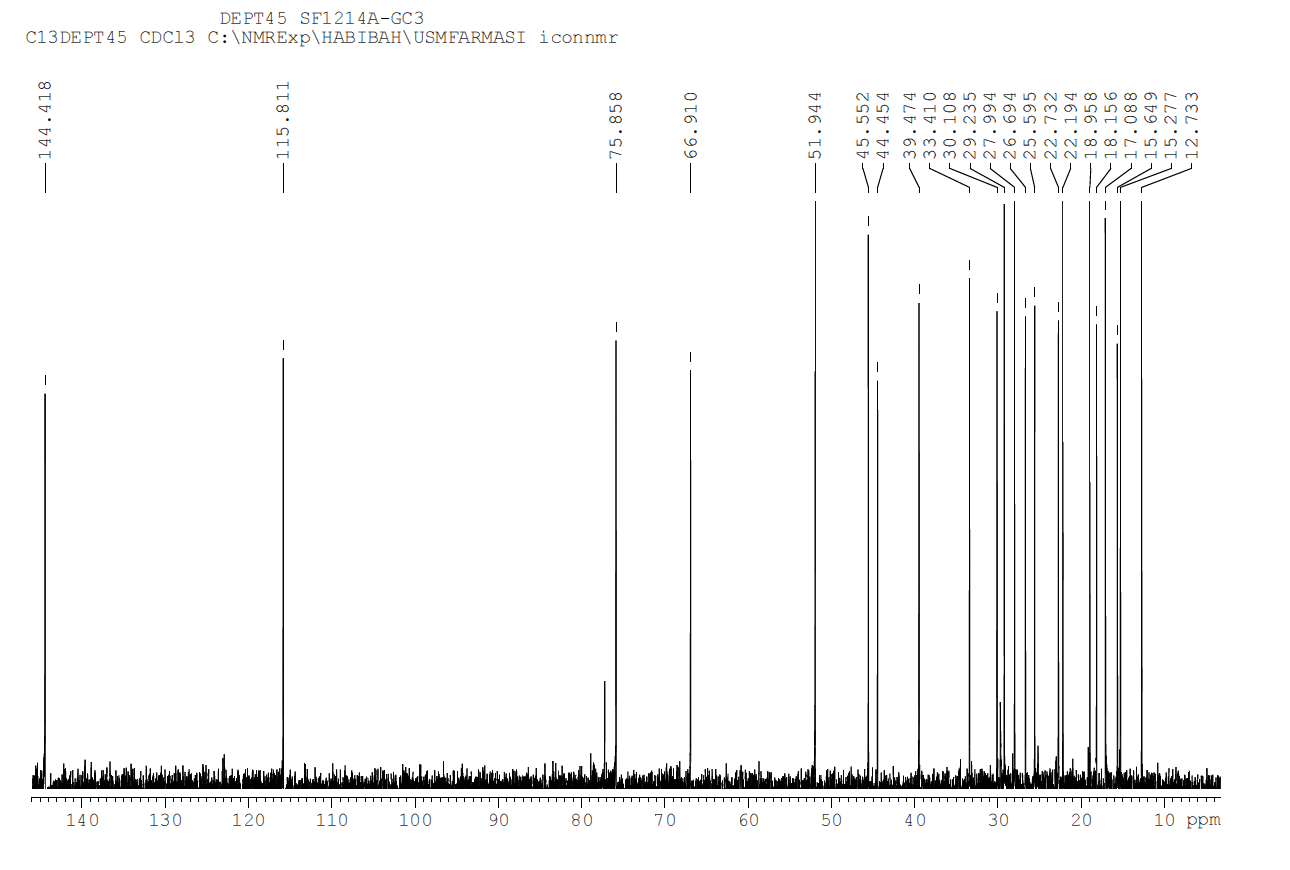
^

^13^C and DEPT45-NMR (top to bottom) spectrum of GC3 compound that isolated from *G. celebica* Leaves using CDCl_3_ solvent

**GC3: continued**


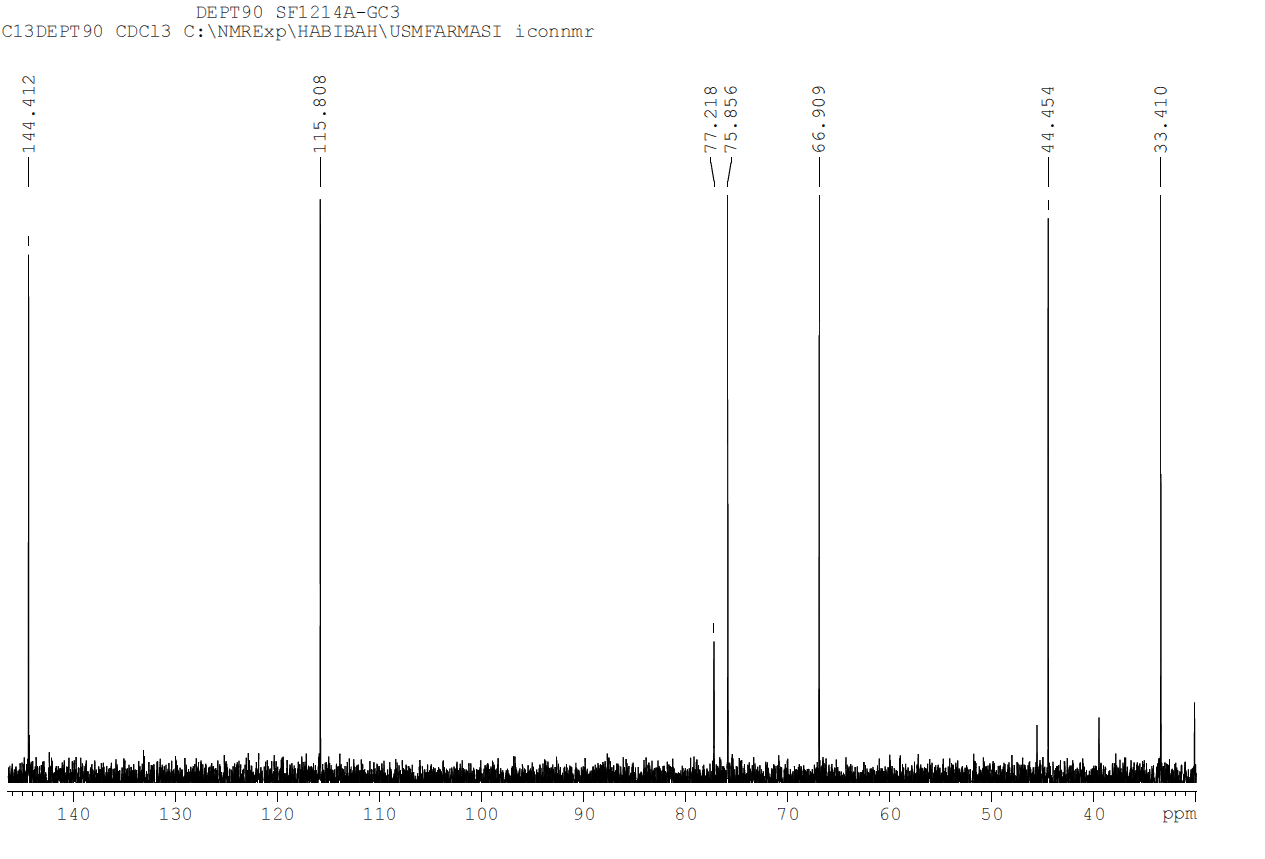


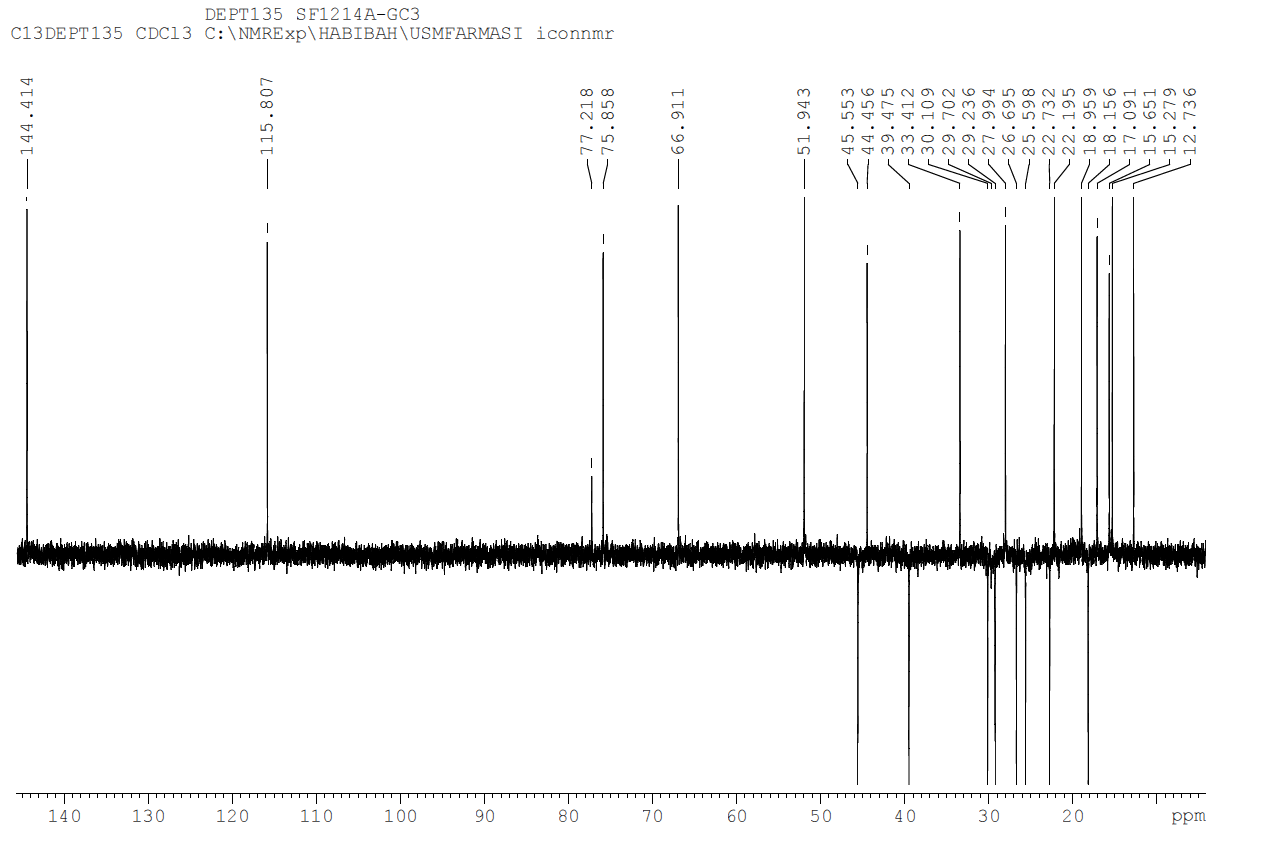


DEPT90 and DEPT135-NMR (top to bottom) spectrum of GC3 compound that isolated from *G. celebica* Leaves using CDCl_3_ solvent

**GC3: continued**

**
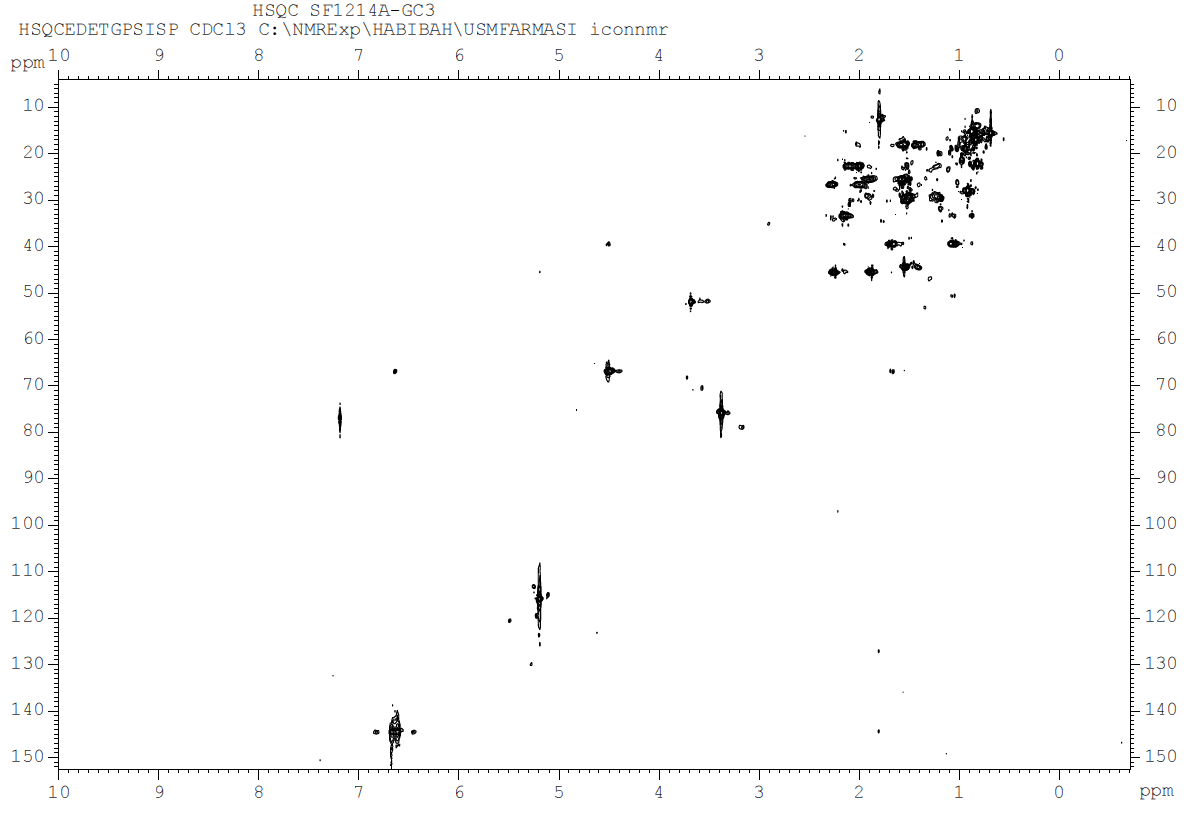
**

**
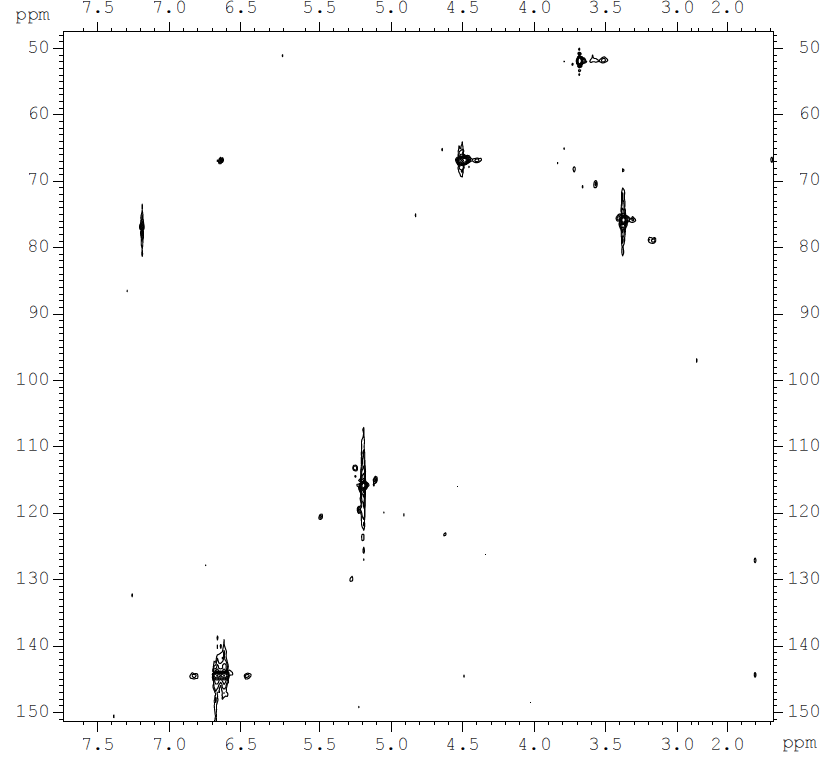

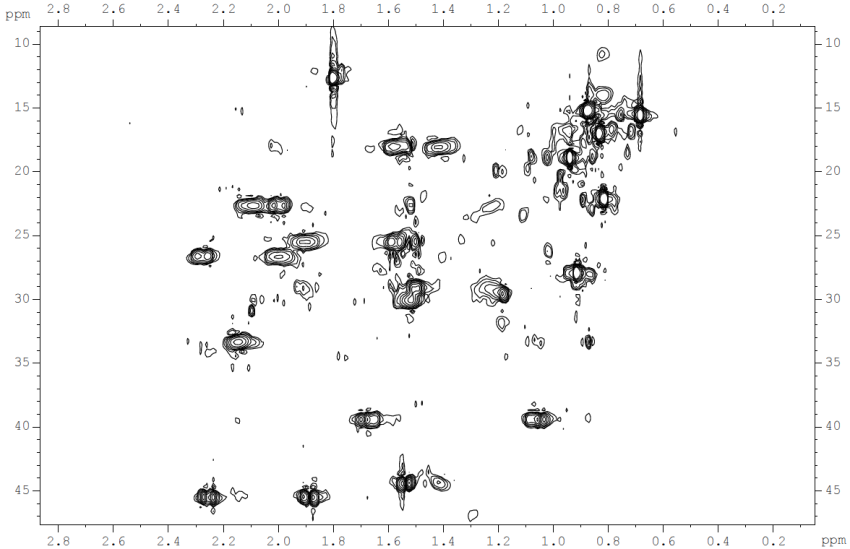
**

2D-HSQC-NMR of GC3 using CDCl_3_ solvent

**GC4: continued**


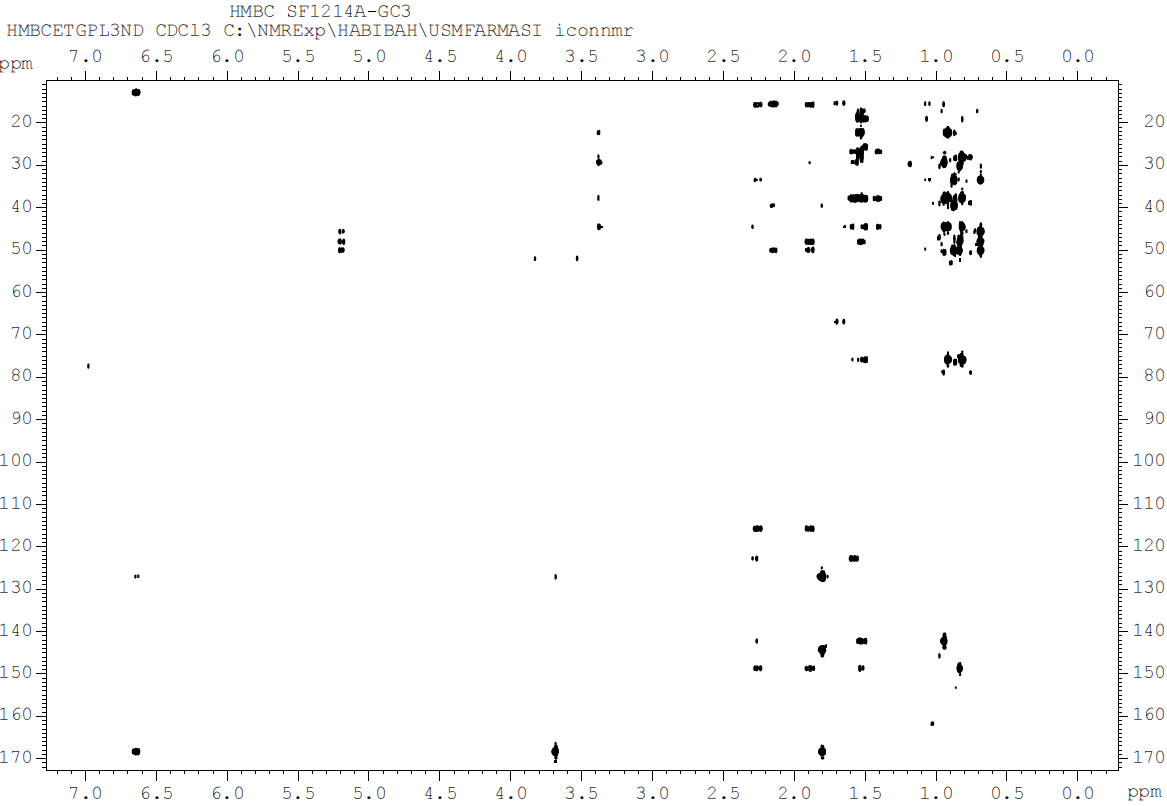


2D-HMBC-NMR of **GC3** using CDCl_3_ solvent


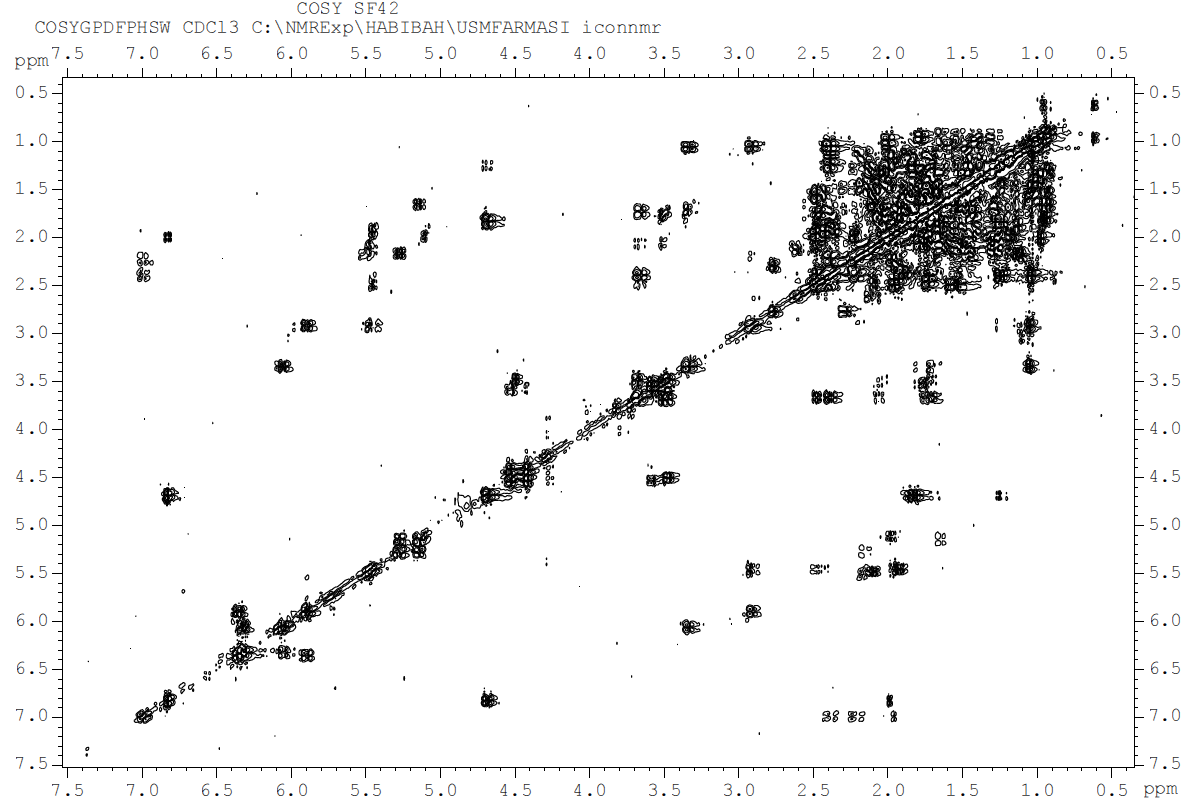


2D-COSY-NMR of GC3 using CDCl_3_ solvent

**Spectroscopy data of GC4**


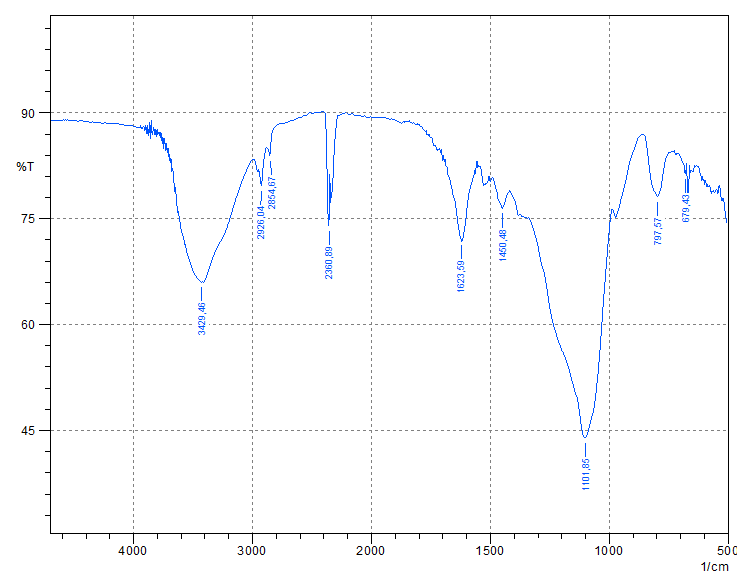


IR spectrum of GC4 compound that isolated from *G. celebica* leaves


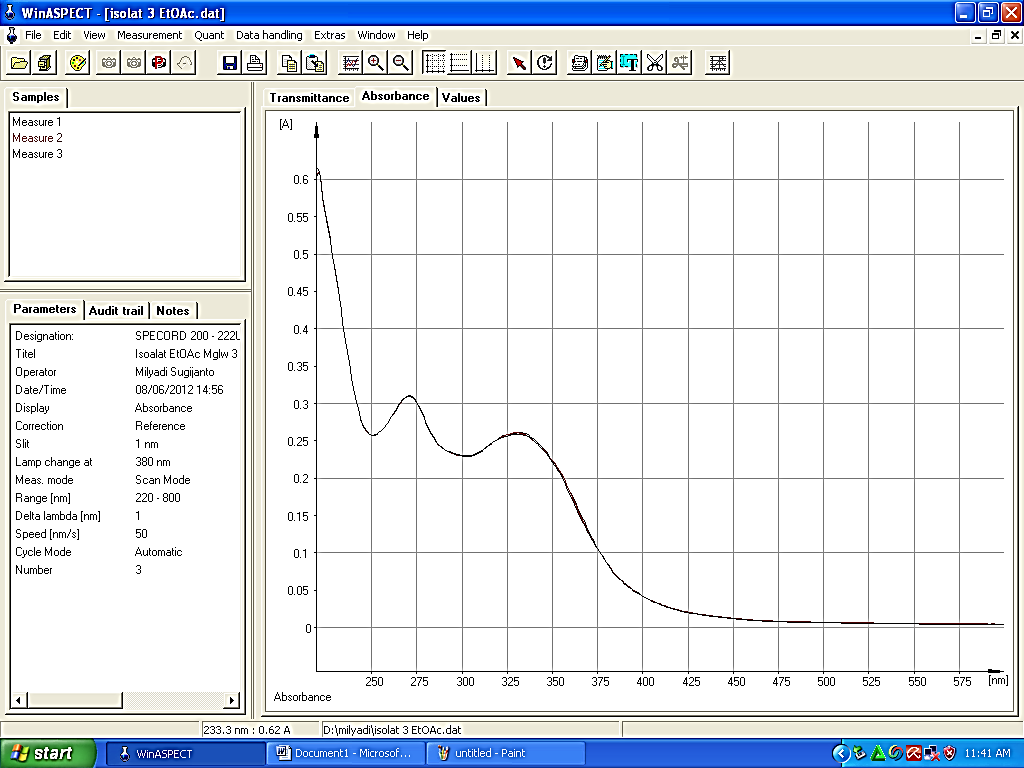


UV spectrum of GC4 compound that isolated from *G. celebica* leaves

**GC4: continued**

**
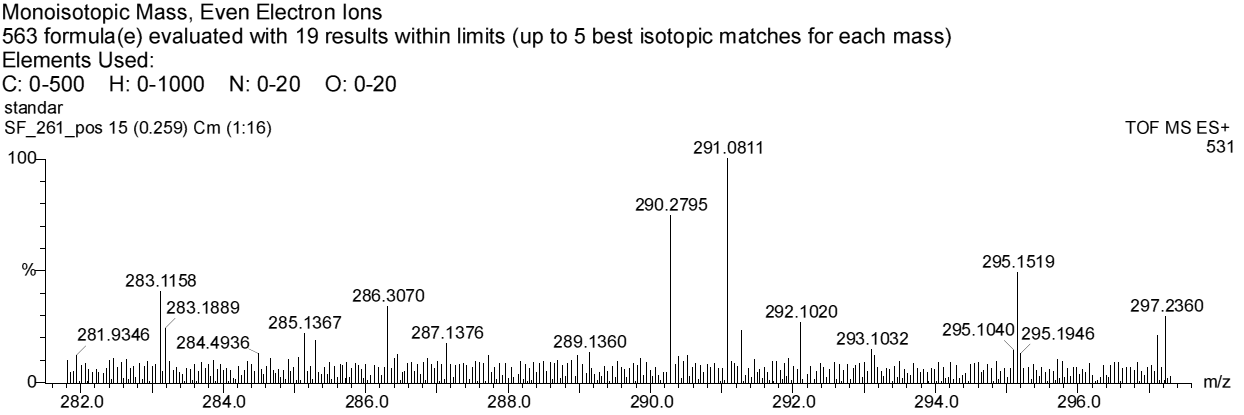
**

**
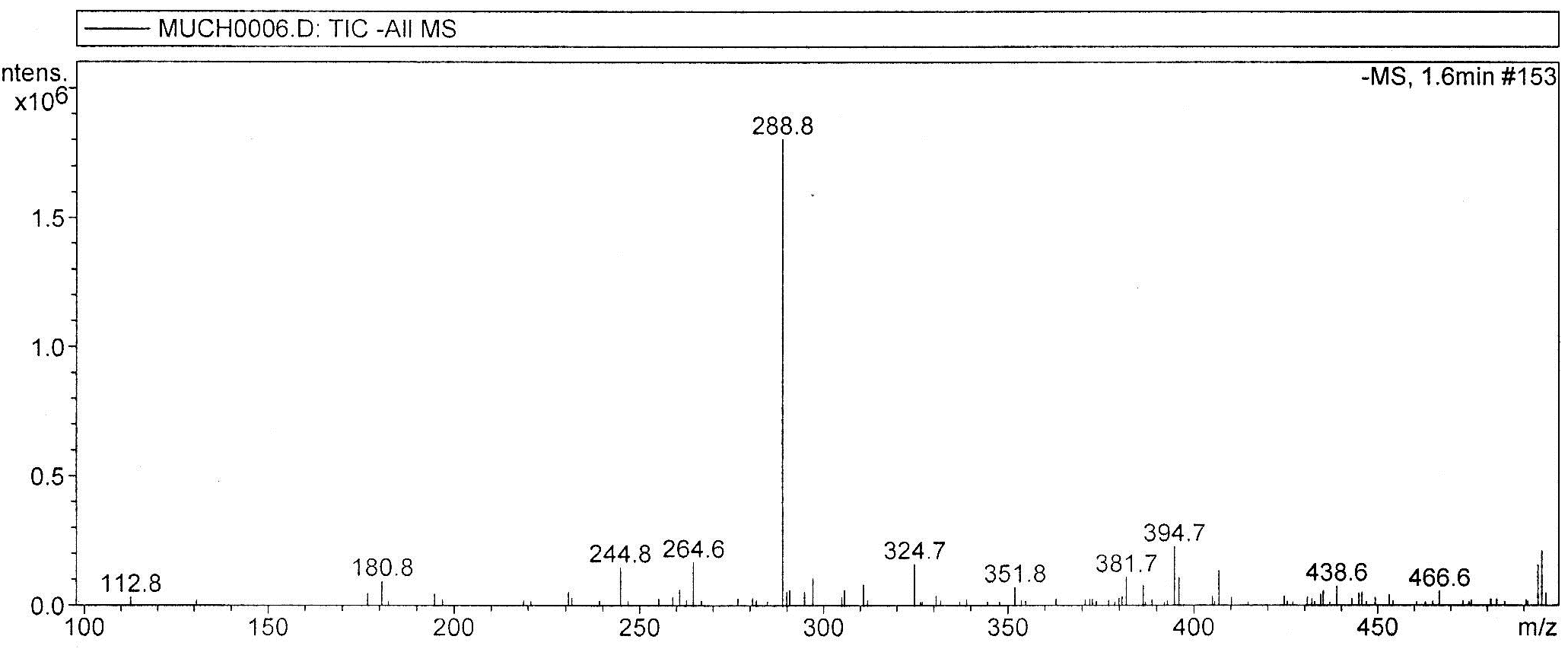
**

Positive Mode [M+H]^+^ using HRESMS-QTOF and Negative Mode [M-H]^+^  (top to bottom) of Mass Spectrum Using ESIMS-Trap-Direct Injection in MeOH of GC4 compound that isolated from *G. celebica* Leaves

**GC4: continued**


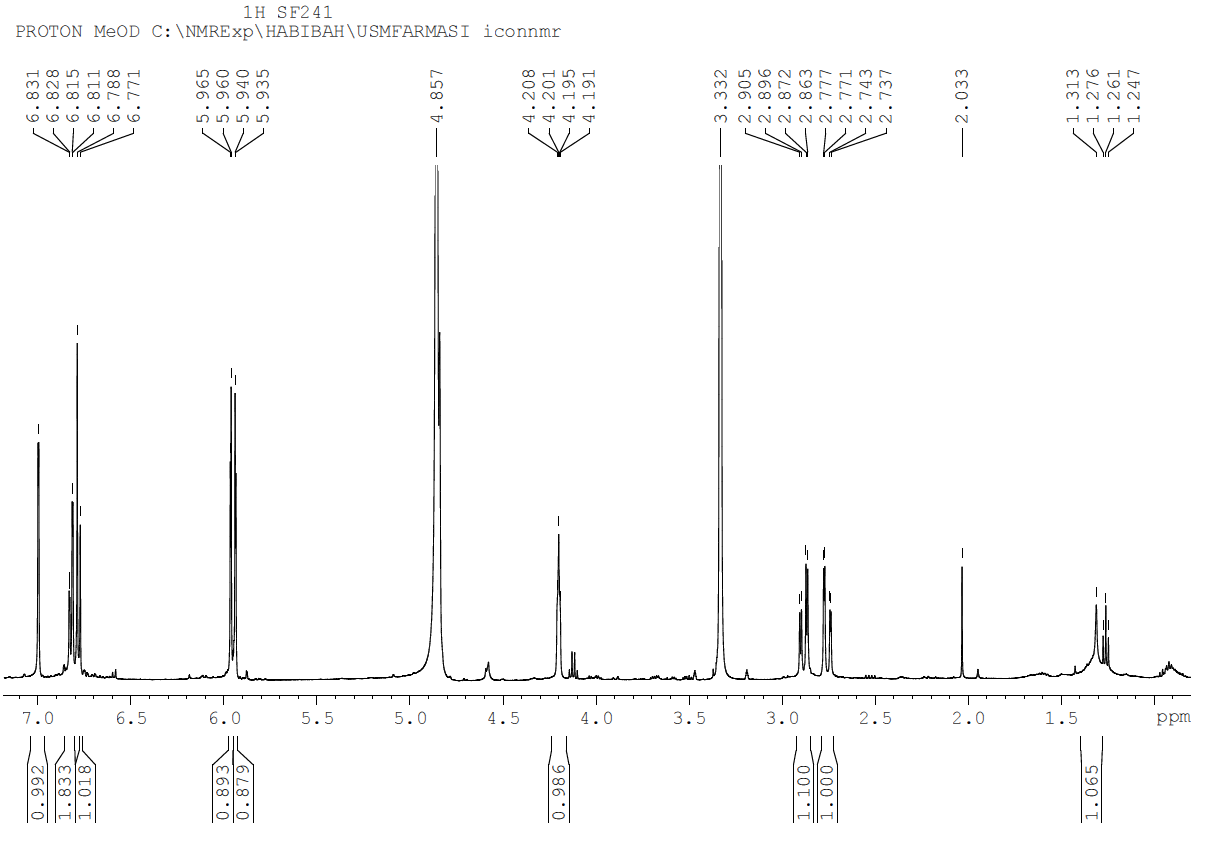


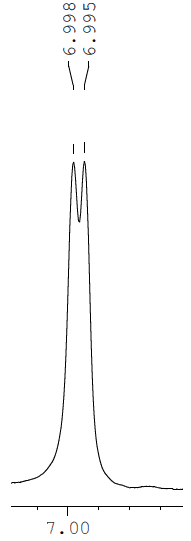

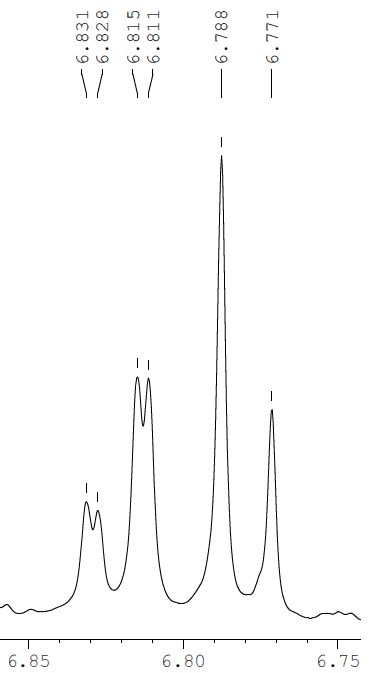

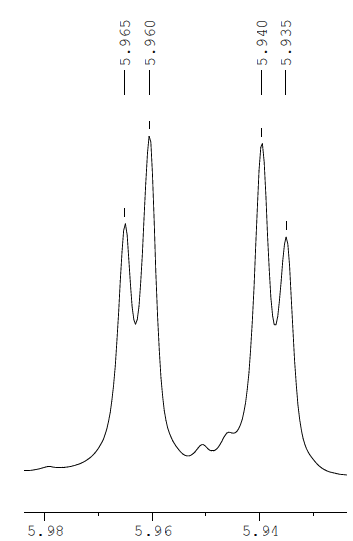

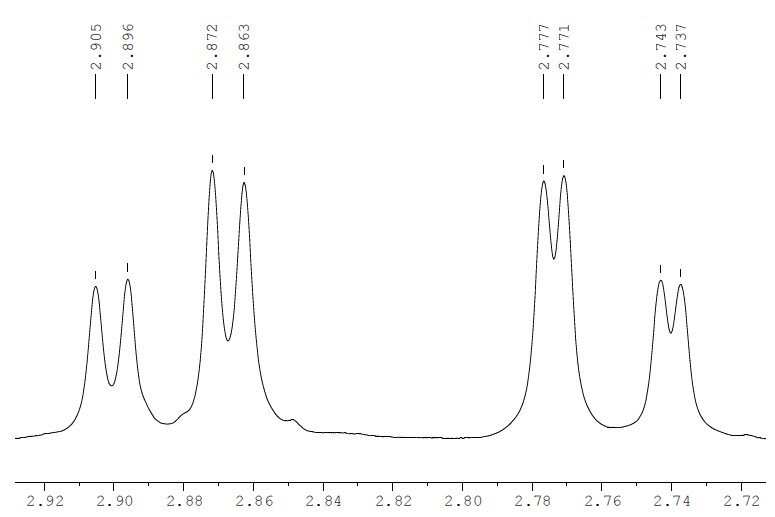


1H-NMR spectrum of GC4 compound that isolated from *G. celebica Leaves*using CD_3_OD solvent

**GC4: continued**

^
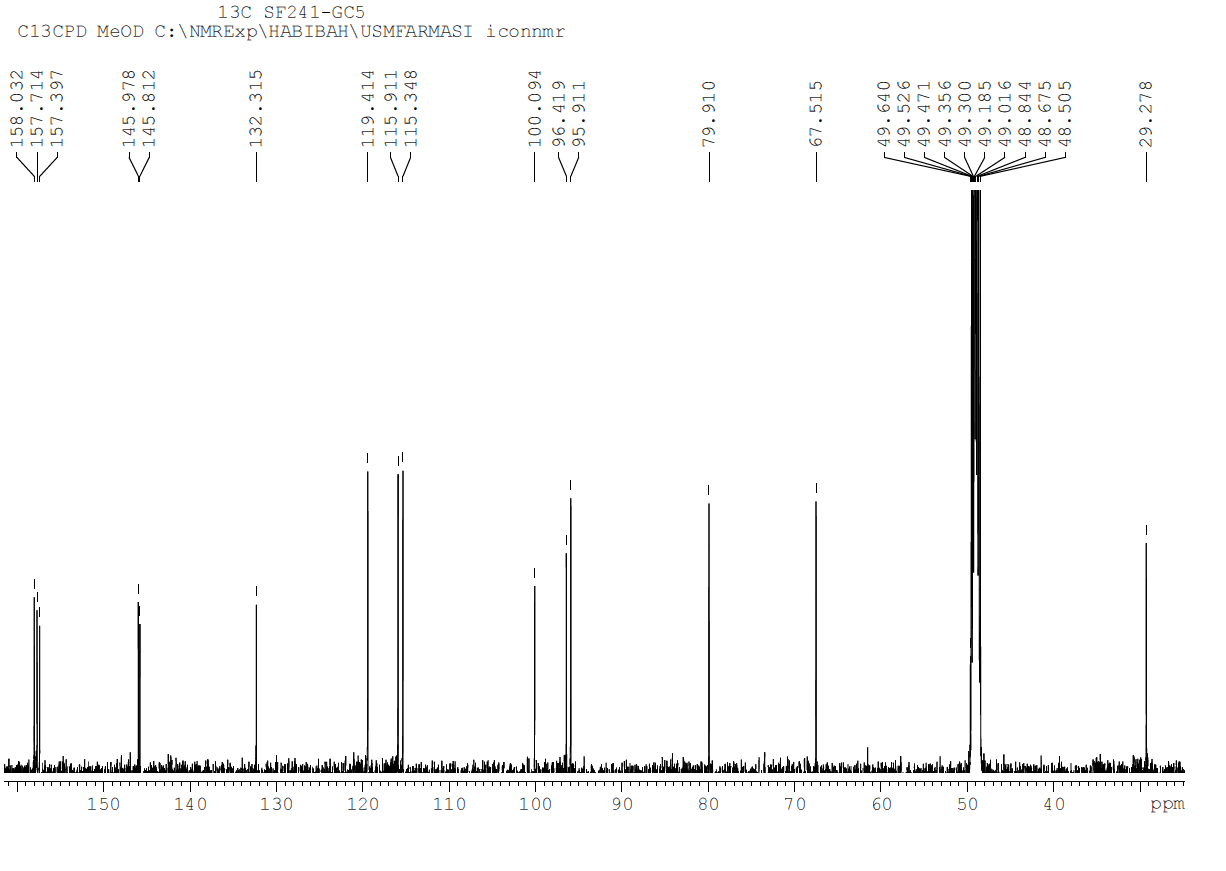
^

^
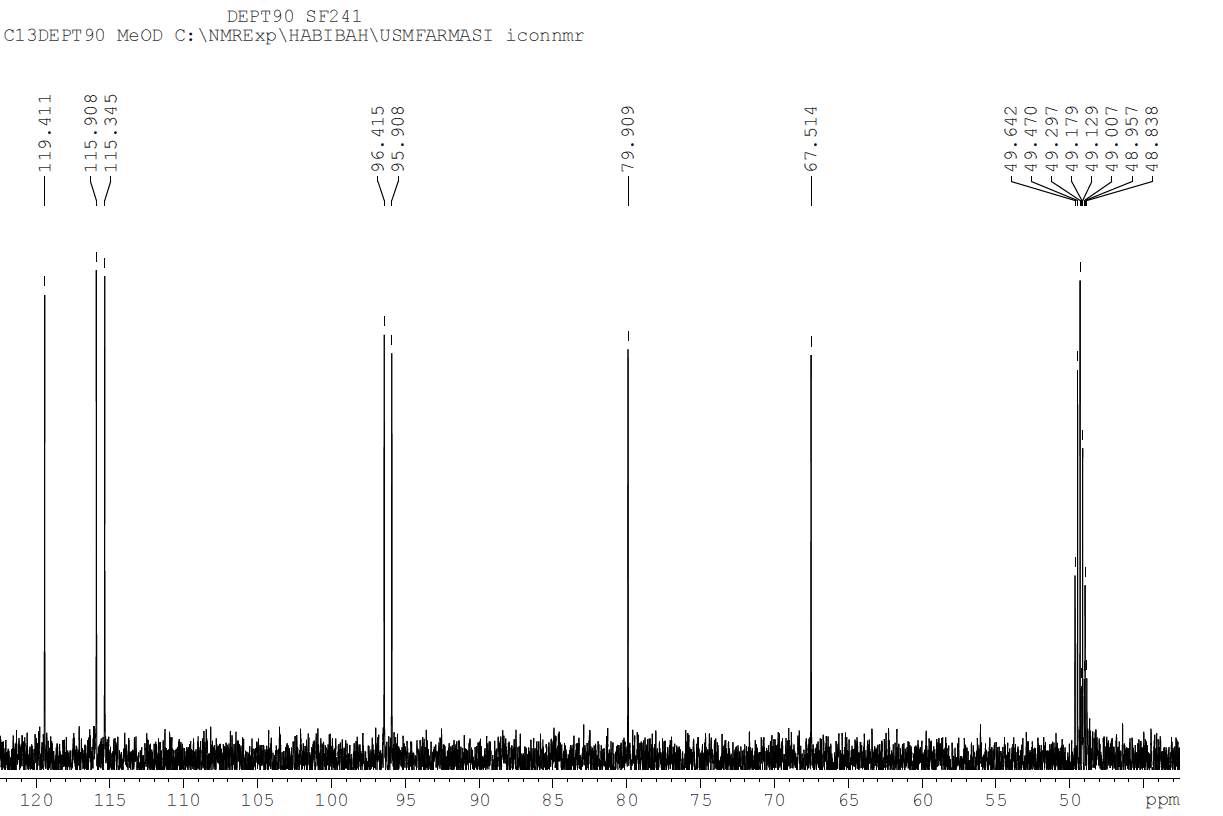
^

^13^C and DEPT45-NMR (top to bottom) spectrum of GC4 compound that isolated from *G. celebica Leaves*using CD_3_OD solvent

**GC4: continued**


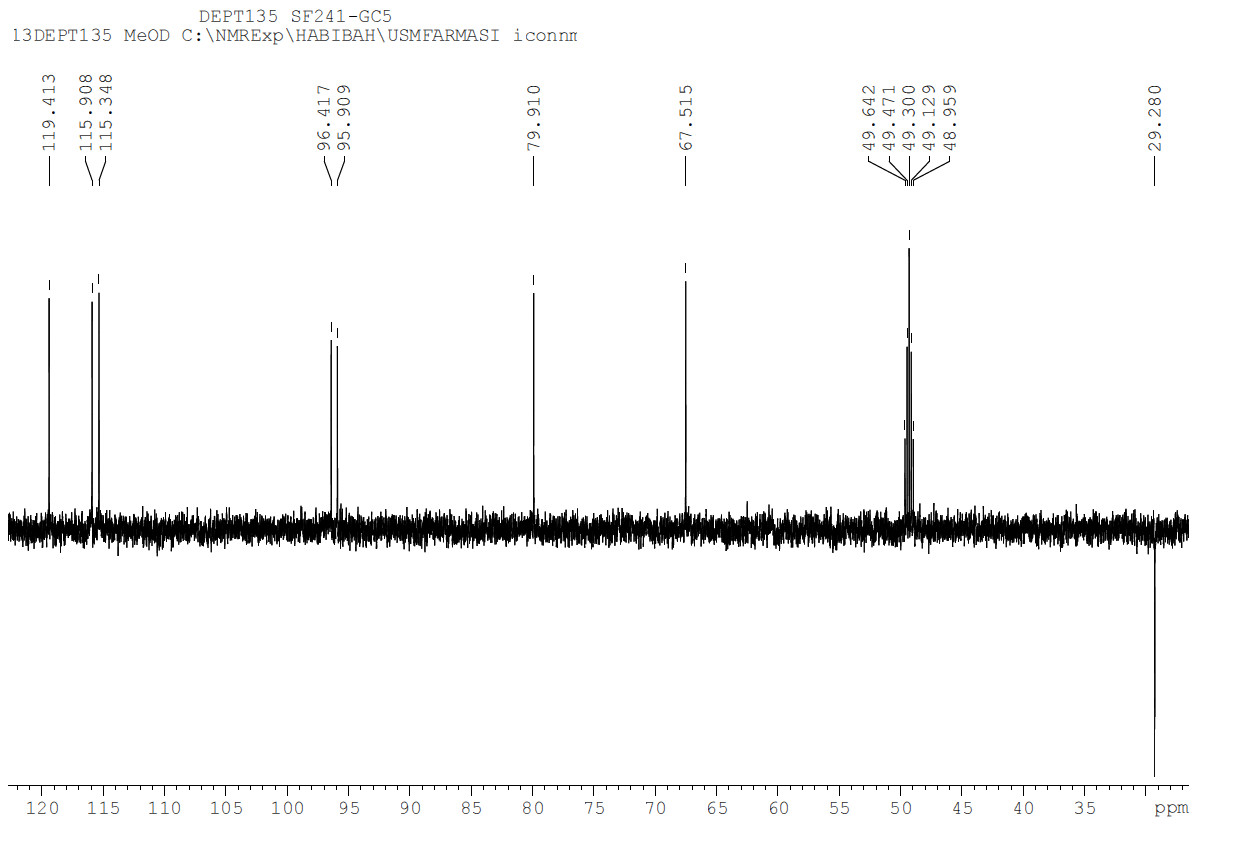


DEPT135-NMR spectrum of GC4 compound that isolated from *G. celebica* Leaves using CD_3_OD solvent

**
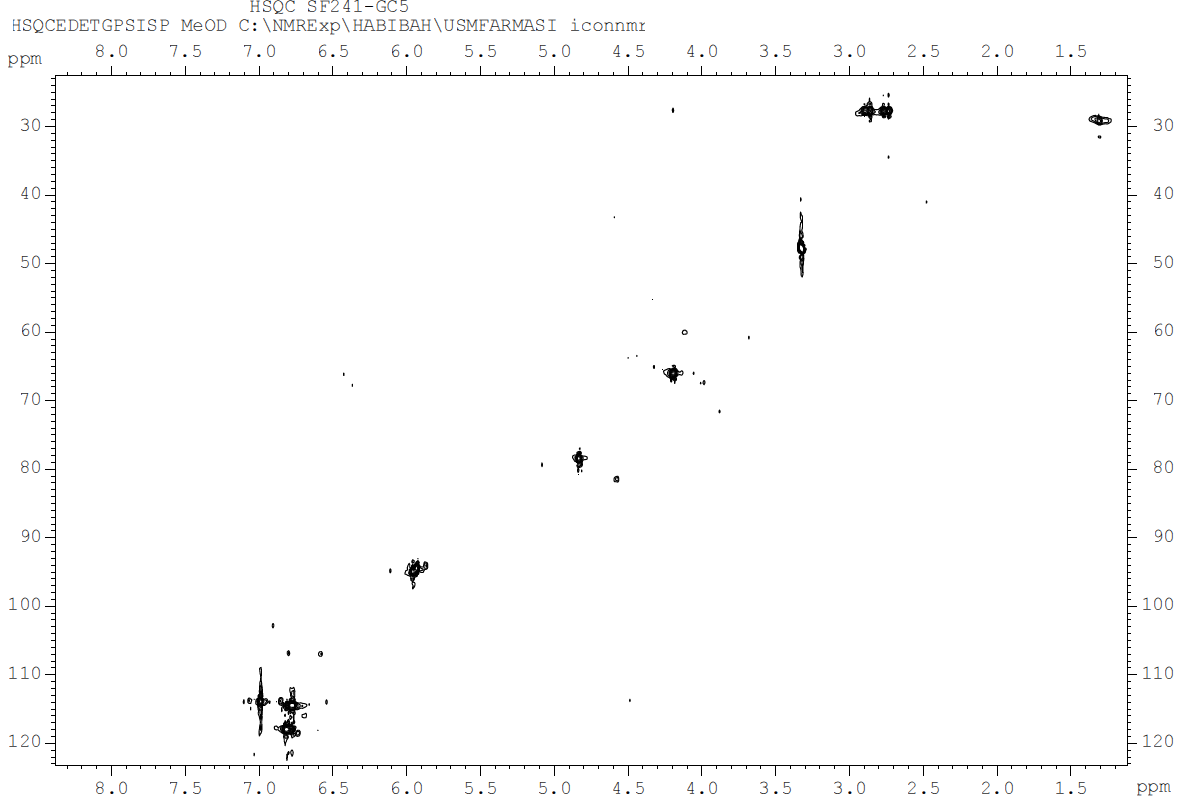
**

2D-HSQC-NMR of GC4 using CD_3_OD solvent

**GC4: continued**


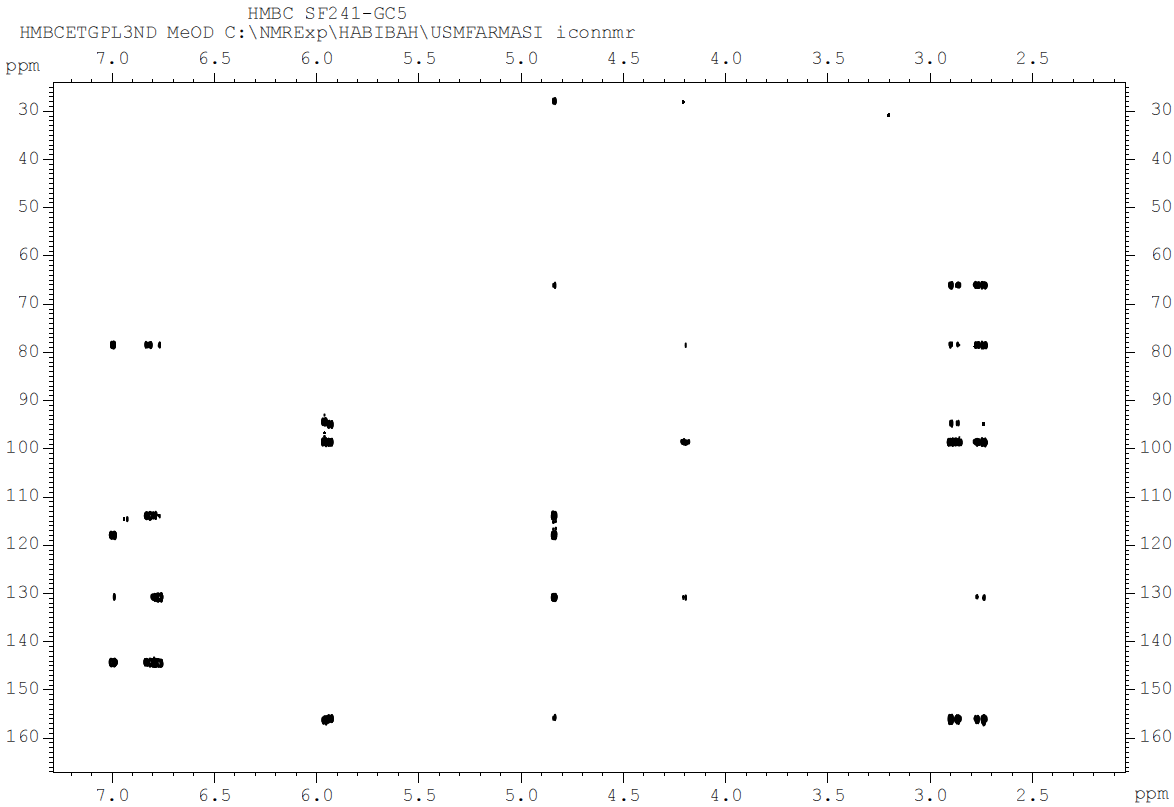


2D-HMBC-NMR of GC4 using CD_3_OD solvent


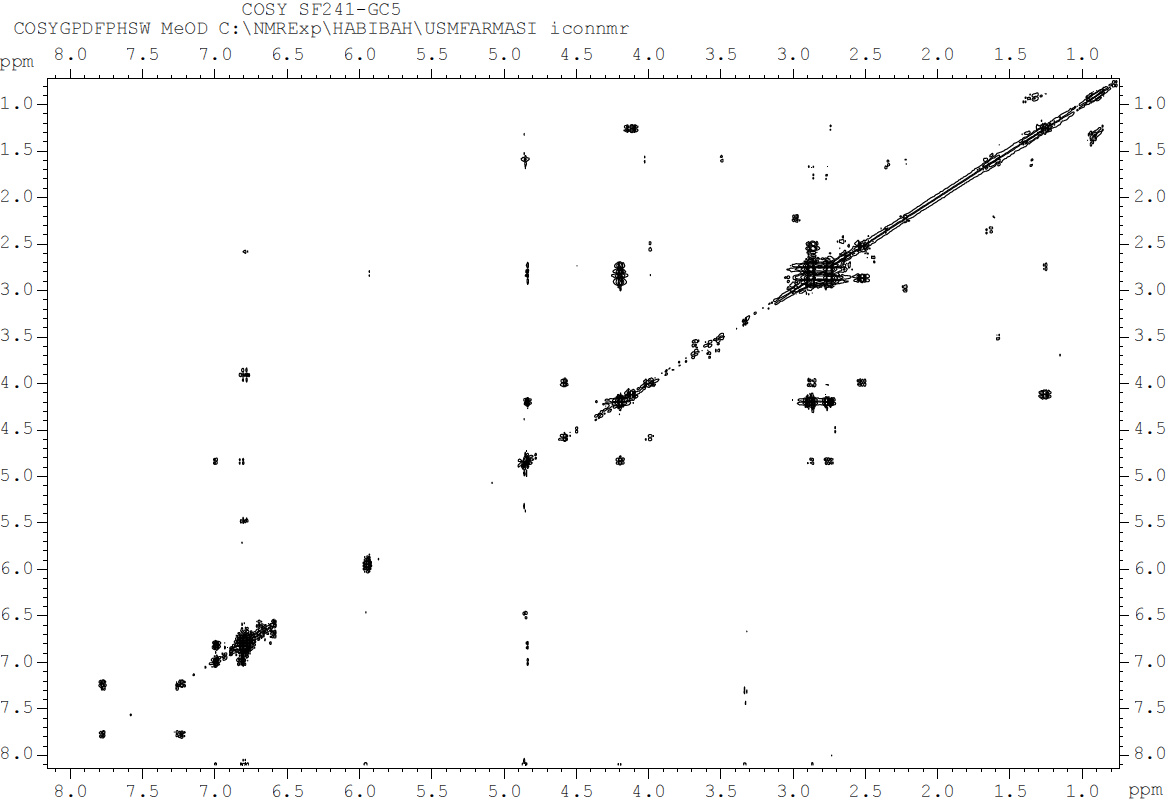


2D-COSY-NMR of GC4 using CD_3_OD solvent
